# Dirac-like cone-based electromagnetic zero-index metamaterials

**DOI:** 10.1038/s41377-021-00642-2

**Published:** 2021-09-30

**Authors:** Yang Li, C. T. Chan, Eric Mazur

**Affiliations:** 1 State Key Laboratory of Precision Measurement Technology and Instrument, Department of Precision Instrument, Tsinghua University, Beijing, China; 2 Department of Physics, Hong Kong University of Science and Technology, Kowloon, Hong Kong China; 3 John A. Paulson School of Engineering and Applied Sciences, Harvard University, Cambridge, MA USA

**Keywords:** Metamaterials, Nanophotonics and plasmonics

## Abstract

Metamaterials with a Dirac-like cone dispersion at the center of the Brillouin zone behave like an isotropic and impedance-matched zero refractive index material at the Dirac-point frequency. Such metamaterials can be realized in the form of either bulk metamaterials with efficient coupling to free-space light or on-chip metamaterials that are efficiently coupled to integrated photonic circuits. These materials enable the interactions of a spatially uniform electromagnetic mode with matter over a large area in arbitrary shapes. This unique optical property paves the way for many applications, including arbitrarily shaped high-transmission waveguides, nonlinear enhancement, and phase mismatch-free nonlinear signal generation, and collective emission of many emitters. This review summarizes the Dirac-like cone-based zero-index metamaterials’ fundamental physics, design, experimental realizations, and potential applications.

## Introduction

In the optical regime, a light wave’s spatial wavelength *λ* = 2πc/*ωn* is at the micro- or nanoscale, resulting in rapid variations in spatial phase over the medium in which light propagates. These variations limit the performance of many optical devices. For example, in guided-wave optics, fibers and waveguides are usually much longer than the spatial wavelength and so they are limited to smooth curvilinear lines without sharp bending, restricting their integration in photonic integrated circuits. In nonlinear optics, the short spatial wavelength makes it challenging to achieve a desired phase relationship between the interacting waves over a long interaction distance, necessitating the use of various phase-matching techniques that restrict the configuration of nonlinear optical elements. In quantum optics, collective emission phenomena require that the linear size of an assembly of atoms or ions be smaller than the emission wavelength, limiting the number of atoms/ions in the assembly and the spatial extent of collective emission phenomena.

In a medium with a refractive index of zero *n* = 0, the effective wavelength becomes infinite, *λ* = 2πc/*ωn* → ∞, and the spatial phase distribution of a propagating wave becomes uniform over the entire medium^1,2^, overcoming many limitations imposed by the short spatial wavelength in the optical regime. In guided-wave optics, a zero-index waveguide can achieve high transmission regardless of shape, significantly improving the prospect of integration of integrated photonic circuits^3–6^. In nonlinear optics, as the spatial wavelength approaches infinity, phase mismatch–free nonlinear generation becomes possible^7^. In quantum optics, collective emission can be realized with an infinite spatial wavelength at the emission frequency with many atoms/ions in a highly extended sample^8^.

Because of causality, an index of zero of a passive medium can only be realized at a single frequency and so in this review, we will focus on the low refractive index near a zero crossing. According to the ultra-low-loss purely dielectric zero-index metamaterials, the bandwidth in which |*n*_eff_| ≤ 0.1 can be as wide as ~5%^9^. Based on the definition of refractive index $n = \sqrt {\varepsilon _{{{\mathrm{r}}}}\mu _{{{\mathrm{r}}}}}$, a near-zero index can be achieved in three ways: permittivity near zero (ENZ), permeability near-zero (MNZ), and permittivity and permeability near-zero (EMNZ)^10–12^. Spatially continuous ENZ can be achieved across a bulk metal near the plasma frequency^2,13,14^. Distinct from the spatially continuous ENZ, macroscopic EMNZ can be realized by traditional metamaterials such as fishnet metamaterials^15^, or by doping an ENZ medium with dielectric dopants^16–20^, or in all-dielectric metamaterials supporting a Dirac-like cone dispersion at the center of the Brillouin zone (“Comparison of Dirac-like cone-based zero index....” section)^21–26^.

Dirac-like cone dispersion is formed by the accidental 3-fold degeneracy of two linear bands with conical dispersion and a quadratic dispersive band (which is flat near *k* = 0) at the center of the Brillouin zone. The group velocities of the two linear dispersive bands change sign at their crossing point. From an effective medium point of view, the change in sign of group velocity implies that the effective refractive index changes sign at the Dirac point, which in turn means that the effective refractive index is zero at the Dirac-point frequency. The zero effective refractive index implies the existence of a quasi-longitudinal solution to the Maxwell equation from a macroscopic point of view, and this is consistent with the existence of the flat band cutting through the Dirac point, giving rise to a 3-fold degeneracy. Using rigorous effective medium homogenization approaches, it can be shown that the metamaterial’s effective permittivity and permeability cross zero simultaneously and linearly at the Dirac-point frequency under certain conditions (“Relationship between a Dirac-cone dispersion at the...” section). The zero effective refractive index of such systems is of special interest as the “double zero” (both permittivity and permeability are zero) means that the impedance can be tuned to match that of the external medium.

Dirac cones typically refer to the conical dispersion of electronic states at the corner of Brillouin zone of materials such as graphene^27^. These electronic states manifest themselves as massless fermions with unique transport properties. Here, we discuss another type of Dirac cone—a Dirac-like cone dispersion at the Brillouin zone center of some photonic crystals. Different from Dirac cones of graphene, which are 2-fold degeneracies at the Dirac point, the Dirac-like cones at zone center are 3-fold degeneracies^21^. And, Dirac-like cones’ transport properties are distinctly different from those of massless fermions. The photonic states of the Dirac-like cone transport as they are traveling in a medium with a refractive index of zero. According to the slope of bands near Dirac point, Dirac cones can be categorized into type-I, II, and III, which show opposite signs of group velocities, identical signs of group velocities and zero group velocities, respectively, at the Dirac-point frequency^28^. Dirac-like cone corresponds to the type-I photonic Dirac cone with an additional flat band.

Time reversed symmetry mandates that isolated bands must have a dispersion that obey *∂ω*/*∂k* = 0 at *k* = 0 and hence Dirac cones with linear dispersion cannot be realigned. However, if there are accidental degeneracies, a Dirac-like cone dispersion with linear dispersion is permitted in some high symmetry lattices. If an effective medium theory can be applied to those systems, the effective *ε*(*ω*) and *μ*(*ω*) pass through zero simultaneously at the Dirac-point frequency where impedance is determined by the ratio of *∂μ*_eff_/*∂ω* and *∂ε*_eff_/*∂ω* and can be tuned to match that of the input medium (“Relationship between a Dirac-cone dispersion at the...” section). The group velocity of the modes on the Dirac-like cone can likewise be tuned by changing the microstructure topology. In contrast, an ENZ or MNZ material has near-zero group velocity and extreme impedances when the material is lossless and spatially unbounded^29^. Such extreme impedances can be tuned to finite by engineering the height of a waveguide filled with ENZ or MNZ medium^3,30^.

In Dirac-like cone-based zero-index metamaterials (DCZIMs), the two curl equations of the Maxwell equations, which couple electric and magnetic phenomena, become zero $\nabla \times \vec E = 0,\nabla \times \vec H = 0$, decoupling the average electric and magnetic fields over the metamaterial. EMNZ also leads to a zero wavevector $\left| {\vec k} \right| = \omega n/c\mathop { \to }\limits^{n = 0} 0$, corresponding to a momentum with zero amplitude and undefined direction. The zero wavevector results in an infinite spatial wavelength $\lambda = 2\pi /\left| {\vec k} \right|\mathop { \to }\limits^{\left| {\vec k} \right| = 0} \infty$ and a zero spatial phase $\vec k \cdot \vec r\mathop { \to }\limits^{\left| {\vec k} \right| = 0} 0$, leading to a uniform spatial phase distribution throughout the metamaterial^31^. This phenomenon can be understood from the viewpoint of phase velocity: the wavefront is able to travel through the metamaterial instantaneously, indicating an infinite phase velocity $v_{{{\mathrm{p}}}} = \omega /\left| {\vec k} \right|\mathop { \to }\limits^{\left| {\vec k} \right| = 0} \infty$. However, DCZIMs still show an energy velocity lower than the velocity of light in a vacuum, satisfying causality. To efficiently excite the zero-index modes of DCZIMs, the incident plane wave has to satisfy the normal incidence condition. Oblique incidence at certain angles excites the “flat band” of the Dirac-like cone (“Homogenization of DCZIMs” section), which does not correspond to zero-index behavior^32,33^. As summary, in a DCZIM excited by a normally incident plane wave, an electromagnetic wave propagates in all directions with an infinite phase velocity, whose corresponding electric and magnetic fields oscillate in unison throughout the metamaterial.

This review provides an overview of the significant progress in the fundamental physics, design, experimental implementations, and potential applications of photonic DCZIMs. We first explain the relationship between a Dirac-cone dispersion at the center of the Brillouin zone and EMNZ. We then analyze the homogenization of DCZIMs as bulk media with effective constitutive parameters. We note that the zero-index materials are realized using dielectric photonic crystals. As the conical dispersion is at *k* = 0 and the dispersion originates from lowest order resonances, effective medium theories can be rigorously applied to extract effective constitutive parameters^34^. In this sense, we can call the photonic crystal a metamaterial even though we are not considering the *ω* → 0 limit. Then, we compare the optical properties of DCZIMs with those of other mechanisms for achieving a near-zero refractive index. We review the design of DCZIMs for various zero-index properties. We then introduce several experimental realizations of DCZIMs in the form of either bulk metamaterials (out-of-plane configuration) or on-chip metamaterials (in-plane configuration). We summarize DCZIMs’ potential applications in electromagnetic waveguides, free-space wave manipulation, metrology, nonlinear optics, lasers, and quantum optics. We finally discuss the pros and cons of DCZIMs in comparison with other mechanisms for achieving zero index and envision DCZIMs’ future development in applications in optical interconnects, nonlinear optics, lasers, and quantum optics.

A good recent review provided a comprehensive summary of the fundamental physics of homogeneous zero-index materials, history of DCZIMs from the metallic EMNZ metamaterials to the all-dielectric EMNZ metamaterials, and challenges of DCZIMs^35^. In contrast, our review focuses on the fundamental physics of DCZIMs, comparison of DCZIM with zero-index materials based on volume plasmon, fishnet metamaterials and doped ENZ medium, classification of DCZIMs, demonstration of optical DCZIMs, and promising potential applications of DCZIMs. In addition to photonic metamaterials, the concept of DCZIMs has already been adopted by other kinds of metamaterials including acoustic^36–47^ and elastic metamaterials^48,49^.

## Relationship between a Dirac-cone dispersion at the center of the Brillouin zone and an impedance-matched zero index

A Dirac-like cone dispersion at the center of the Brillouin zone is a necessary but not sufficient condition to achieve the EMNZ behavior^50^. According to rigorous effective medium theories^34^, only a Dirac-like cone dispersion consisting of monopole and dipole modes at the center of the Brillouin zone at a long wavelength can correspond to the EMNZ behavior^50^. Such a condition can be met by a square or triangular lattice of 2D dielectric pillars whose index contrast between pillar and background matrix is high enough under transverse magnetic (TM) polarization. Many other structures^31,51–55^, such as a square lattice of air holes in a dielectric matrix showing a Dirac-like cone dispersion consisting of transverse electric (TE) polarized dipole and quadruple modes at the center of the Brillouin zone^51^, cannot fully satisfy the strict condition of EMNZ. To be consistent with previous literature, we still treat these structures as “Dirac-like cone-based EMNZ metamaterials” in this review despite the fact that they can only show phase-free propagation at the Dirac-point wavelength^31^. To experimentally realize a Dirac-like cone-based metamaterial meeting the strict condition of EMNZ, we can sandwich a square or triangular lattice of 3D dielectric pillars in-between two perfect electrical conductor (PEC) plates with perfect magnetic conductor (PMC) plates as sidewalls^21^. Here, we use a square lattice of 2D dielectric pillars to explain how does a Dirac-like cone dispersion induces an impedance-matched zero index.

**Fig. 1:**
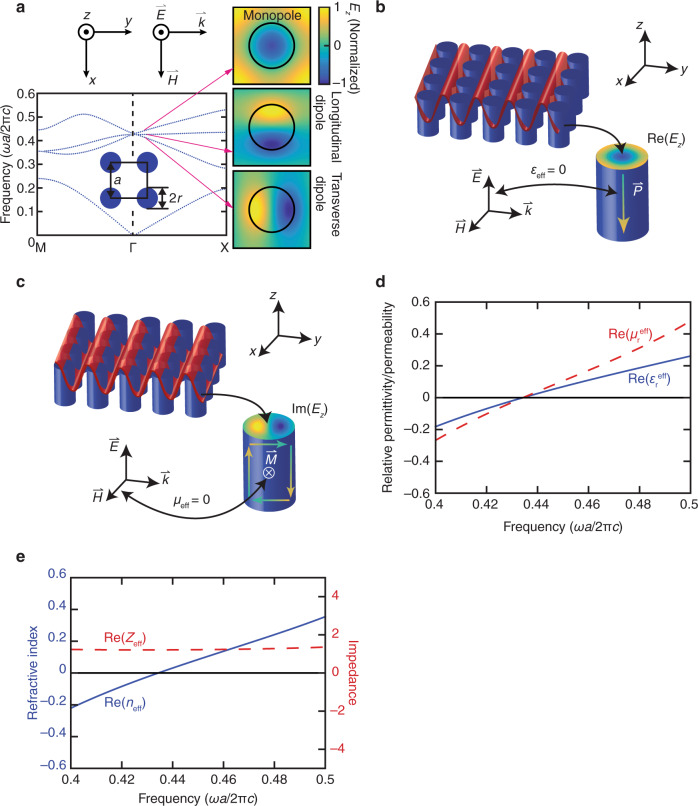
A Dirac-like cone dispersion induces an impedance-matched zero index. **a** Photonic bandstructure of a square lattice of 2D dielectric pillars (inset) and the three modes (right) forming the TM Dirac-like cone dispersion. **b** An electrical monopole mode induces an effective permittivity of zero. **c** A transverse magnetic dipole mode induces an effective permeability of zero. **d** The effective permittivity and permeability as well as **e** the effective refractive index and impedance calculated by an effective medium theory^34^

As shown in Fig. 1a, at the center of the photonic bandstructure of a square lattice of 2D dielectric pillars, a photonic Dirac-like cone is induced by the accidental degeneracy of an electric monopole mode, a transverse magnetic dipole mode, and a longitudinal magnetic dipole mode, in which the averaged magnetic field is parallel to the *k*-vector^21^. For a TM-polarized Dirac-like cone with an electric field polarized along the pillar axis (*E*_*z*_), the longitudinal magnetic dipole mode cannot be excited because the corresponding magnetic-field component is missing in TM-polarized excitation. The monopole mode and transverse magnetic dipole mode correspond to the real and imaginary parts of *E*_*z*_, respectively, thus relating the two orthogonal modes in the photonic bandstructure to the two orthogonal components of the time-harmonic *E*_*z*_ in the spatial domain. As time varies, the distribution of *E*_*z*_ over the metamaterial oscillates between a monopole mode and a transverse dipole mode. Here, we use a simple model in Fig. 1b, c to explain how these monopole modes and transverse dipole modes induce zero permittivity and permeability.

As shown in Fig. 1b, Re(*E*_*z*_) (monopole mode) corresponds to a sinusoidal wave with troughs in the middle of the pillars, resulting in a polarization $\vec P$ with the same amplitude as the applied electric field $\varepsilon _0\vec E$ but in the opposite direction. Such a polarization thus cancels out the applied electric field $\varepsilon _0\vec E + \vec P = 0$. Based on the definition of electric flux $\vec D = \varepsilon _0\vec E + \vec P$, we have $\vec D = 0$. If we treat the metamaterial as a homogeneous bulk medium with an effective permittivity *ε*_eff_, we can express the constitutive relationship as $\vec D = \varepsilon _{{{{\mathrm{eff}}}}}\vec E$. And, because $\vec D = 0$ and the fact that the applied electric field $\vec E \,\ne\, 0$, we can conclude that *ε*_eff_ = 0.

As shown in Fig. 1c, Im(*E*_*z*_) (transverse dipole mode) corresponds to a sinusoidal wave with neighboring crests and troughs on the left and right surfaces of each pillar, respectively, resulting in upward- and downward-pointing polarizations. These polarizations form a loop and induce, according to Faraday’s law, a magnetization $\vec M$ with the same amplitude as the applied magnetic field $\vec H$ but in the opposite direction. Such a magnetization thus cancels out the applied magnetic field $\vec H + \vec M = 0$. Based on the definition of magnetic flux $\vec B = \mu _0\left( {\vec H + \vec M} \right)$, we have $\vec B = 0$. If we treat the metamaterial as a homogeneous bulk medium with an effective permeability *μ*_eff_, we can express the constitutive relationship as $\vec B = \mu _{{{{\mathrm{eff}}}}}\vec H$. And, because $\vec B = 0$ and the fact that the applied magnetic field $\vec H \,\ne\, 0$, we can conclude that *μ*_eff_ = 0.

The combination of a zero effective permittivity and a zero effective permeability yields a finite impedance $$Z = \mathop {{\lim }}\limits_{\begin{array}{*{20}{c}} {\varepsilon _{{{{\mathrm{eff}}}}} \to 0} \\ {\mu _{{{{\mathrm{eff}}}}} \to 0} \end{array}} \sqrt {\mu _{{{{\mathrm{eff}}}}}/\varepsilon _{{{{\mathrm{eff}}}}}} = {{{\mathrm{finite}}}}\;{{{\mathrm{value}}}}$$. This conclusion predicted by the model is confirmed by the effective constitutive parameters (Fig. 1d, e) computed by a rigorous effective medium theory^34^.

## Homogenization of DCZIMs

To verify that it is proper to treat a 2D DCZIM macroscopically as a homogeneous bulk medium with constitutive parameters near the Γ point, we conduct a systematic homogenization analysis considering the homogenization criteria and locality conditions^56,57^. This 2D DCZIM consists of a square array of silicon pillars in a SU-8 background matrix with Dirac-point wavelength at 1550 nm (Fig. 1a). We also compare DCZIMs with traditional zero-index metamaterials from the viewpoint of homogenization.

### Homogenization criteria

The general homogenization criteria of metamaterials require *k*_0_*a*, *ka* ≤ 1, where *a* is the lattice constant, *k*_0_ and *k* are the free-space wavenumber, and the effective wavenumber in the metamaterial, respectively^58^. For the homogenization of 2D photonic crystals, an effective medium theory extends the general homogenization criteria to a region where *ka* ≤ 1 and *k*_0_*a* > 1^34^. Based on this theory, the homogenization of DCZIMs has been investigated rigorously^50^. Here, we provide a less mathematical and more intuitive homogenization analysis of the metamaterial based on the extended homogenization criteria.

**Fig. 2:**
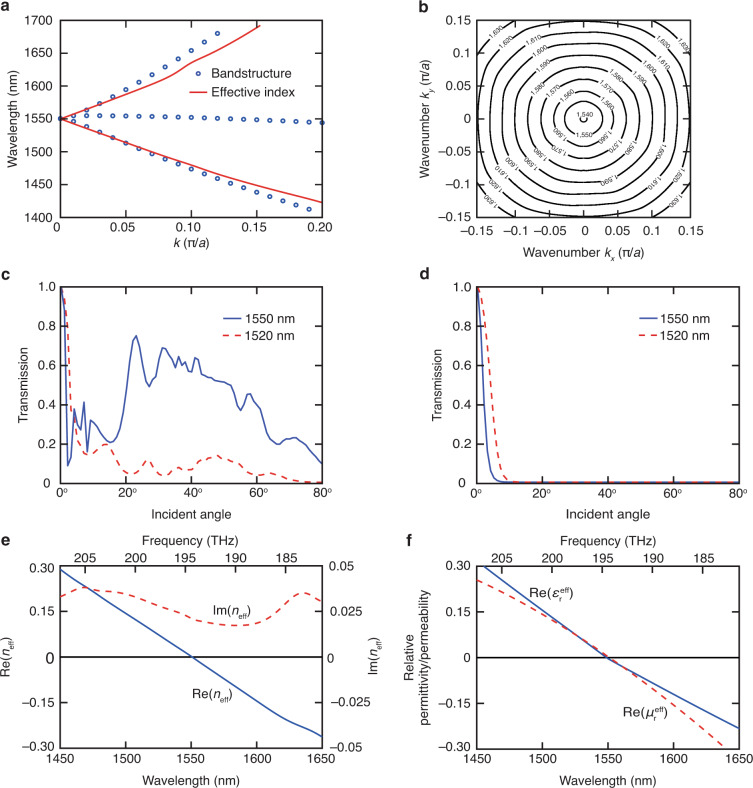
Optical properties of a DCZIM consisting of a square array of silicon pillars in a SU-8 background matrix. **a** Comparison of band structures computed by determining the angular frequencies as a function of wave vector for all the Bloch modes (blue dots) and by *k* = *n*_eff_*ω*/c with the retrieved effective index *n*_eff_ (red curve). **b** Iso-frequency contours. Angle-dependent transmissions of the metamaterial (**c**) and its bulk homogenized model (**d**). Retrieved effective index (**e**) and effective permittivity and permeability (**f**)

We first treat the metamaterial as an infinite array. Because the Dirac-like cone appears near the Γ point (Fig. 1a) where the effective wavenumber *k* approaches zero, this metamaterial satisfies the criterion *ka* ≤ 1 in the region near Dirac point. To quantitatively evaluate the accuracy of the effective medium approach in this region, we compare band structures computed by a macroscopic method regarding the metamaterial as a homogeneous bulk medium (using the retrieved effective index *n*_eff_ and the relationship *k* = *n*_eff_*ω*/c) and a microscopic method treating the metamaterial as an infinite array (photonic bandstructure). The effective constitutive parameters are retrieved from the simulated complex reflection and transmission coefficients of the metamaterial^57,59^. In this retrieval algorithm, the multiple branches of the effective refractive index are selected under the guidance of the effective phase index computed from the phase of the simulated near-field *E*_*z*_ within the metamaterial^57^. As shown in Fig. 2a, the two band structures agree with each other well in the ranges 0 < *k* < 0.1 and 0 < *k* < 0.04 below and above the Dirac point, respectively. Hence, it is a good approximation to treat the infinite array as a homogeneous bulk medium in these regions. This conclusion is valid for all propagation directions near the Dirac point in the Brillouin zone because the metamaterial is almost isotropic near the Dirac point (Fig. 2b).

To study the homogenization of a finite-sized metamaterial, we then compare the angle-dependent transmissions of the metamaterial (Fig. 2c) and its bulk model (Fig. 2d) at an incident angle ranging from 0^o^ to 80^o^ at the Dirac-point wavelength. Both metamaterial and bulk model show high transmission at normal incidence, indicating a strong angular selectivity. Because of the zero critical angle of the zero-index media, we only expect transmission at normal incidence. The undesired transmission of the metamaterial at larger incident angles is due to the fact that the oblique incident TM-polarized light can excite a longitudinal dipole mode which does not correspond to a zero index^21^. This undesired transmission is decreased at a wavelength shorter than the Dirac-point because the longitudinal dipole mode does not exist at this wavelength (Fig. 2a). Hence, the metamaterial can only be treated as a homogeneous bulk zero-index medium for normal incidence at the boundary^32,33^.

### Locality conditions

Effective constitutive parameters of metamaterials have to satisfy the locality conditions: passivity, causality, and isotropy^60^. Fig. 2e shows that the medium is passive due to the fact that Im(*n*_eff_) > 0 near the Dirac-point wavelength. As shown in Fig. 2f, both *∂*(Re(*ε*_r_^eff^))/*∂ω* > 0 and *∂*(Re(*μ*_r_^eff^))/*∂ω* > 0 near the Dirac-point wavelength, indicating that the medium satisfies the basic causality conditions represented by the Kramers–Kronig relations^61^. The fact that the retrieved effective constitutive parameters satisfy the conditions of passivity and causality validates the effectiveness of our homogenization procedure. Furthermore, the almost circular iso-frequency contours in a ~100-nm bandwidth around the Dirac point (Fig. 2b) suggests that the metamaterial is isotropic, leading to an effective refractive index of zero in nearly all propagation directions within the array.

### Comparison between DCZIMs and traditional zero-index metamaterials

The traditional zero-index metamaterial structures typically originate from metallic thin wire array and metallic resonators array^62^. The metallic wire array is used to reduce the plasma frequency to the desired ENZ frequency^63^ while the metallic resonators array is used to achieve a magnetic resonance, leading to a zero permeability at the magnetic plasma frequency^64^. By engineering periodic structures which combine metallic thin wires and metallic resonators in the form of fishnet, the plasma frequency could overlap with magnetic plasma frequency, resulting in an EMNZ behavior^15^ . On the other hand, DCZIMs consisting of 2D dielectric cylinders array achieve EMNZ behavior based on Mie theory. By engineering the period and radius of the array (Fig. 1a), these metamaterials can overlap the plasma frequency of the lowest-order resonance of the Mie monopole scattering coefficient with that of the lowest-order resonance of the Mie dipole scattering coefficient, leading to an EMNZ behavior (Fig. 2f).

EMNZ wavelength of an optical fishnet metamaterial is restricted by the low permittivity of the dielectric material in-between metallic layers^7,15,65–67^. Such a low permittivity decreases the magnetic polariton coupling between adjacent metallic layers, which can be modeled by two coupled resonant LC circuits^67^. A smaller coupling leads to a smaller effective mutual inductance between the two coupled LC circuits, resulting in a higher resonant frequency and a shorter EMNZ wavelength of the metamaterial. On the other hand, due to the low-index contrast between the inclusion and the background matrix composing optical DCZIMs, these metamaterials usually cannot achieve Mie resonances and the corresponding EMNZ behavior in the long-wavelength regime. So far, the highest index contrast of optical DCZIMs is achieved between silicon inclusion and air background matrix—3.47, leading to an EMNZ behavior at *k*_0_*a* ≈ 3.4^21^. Hence, it is challenging for both fishnet metamaterials and DCZIMs to satisfy the homogenization criterion *k*_0_*a* ≤ 1. However, because zero-index modes of both fishnet and DCZIMs are near the *k* = 0 point of the dispersion diagram, both metamaterials can satisfy the homogenization criterion *ka* ≤ 1.

Both fishnet and DCZIMs can meet the locality conditions of passivity and causality. The DCZIMs are by nature isotropic while fishnet metamaterials are anisotropic^15,65,66^.

## Comparison of Dirac-like cone-based zero index with spatially continuous zero index provided by volume plasmon, and zero indices achieved by fishnet metamaterial and photonic doping

To understand the underlying physics of the Dirac-like cone-induced zero index, we compare its optical properties with those of spatially continuous zero index provided by bulk metals—volume plasmons and macroscopic zero index achieved by fishnet metamaterials and by doping an ENZ medium with dielectric dopants.

When a uniform electric field is applied to a thin slab of metal at the plasma frequency *ω*_p_, all the electrons in the metal slab will show in-phase oscillation in the longitudinal direction (i.e., the wavevector direction). Such a collective longitudinal oscillation of free electrons is called a volume plasmon. It leads to the cancellation of the left two terms of the wave equation $\mathop{k}\limits^{\rightharpoonup} \left( {\mathop{k}\limits^{\rightharpoonup} \cdot \mathop{E}\limits^{\rightharpoonup} } \right) - k^2\mathop{E}\limits^{\rightharpoonup} = - \varepsilon \omega ^2\mathop{E}\limits^{\rightharpoonup} /c^2$, resulting in a zero permittivity and hence a refractive index of zero. As a result, based on the constitutive relationship $\mathop{D}\limits^{\rightharpoonup} = 0 = \varepsilon _0\mathop{E}\limits^{\rightharpoonup} + \mathop{P}\limits^{\rightharpoonup}$, polarization within the metal slab cancels out the applied electric field. The longitudinal nature of the volume plasmon prevents it from coupling to transverse electromagnetic waves, decoupling the time-harmonic electric and magnetic fields. This phenomenon can also be explained by zero permittivity. The zero permittivity and refractive index provided by the volume plasmon is continuous over a volume. Such a zero refractive index can also be observed in the dispersion diagram, in which the volume plasmon corresponds to a zero wavenumber at *ω*_p_.

Although the longitudinal nature of the volume plasmon prevents it from coupling to transverse waves and disables almost all the potential light-matter interactions within the bulk metal, the bulk metal can still show ENZ behavior around the *ω*_p_. At frequencies *ω* > *ω*_p_, the bulk metal has a positive refractive index and behaves like transparent dielectrics, enabling the propagation of transverse waves inside the metal. In this region, the metal’s dispersion curve is inside the radiation continuum above the light line, indicating a good coupling to free space via transverse waves. Although most metals’ *ω*_p_ are in the ultraviolet regime, some other materials’ *ω*_p_ are in various frequency regimes. For example, some doped semiconductors, such as indium tin oxide, have *ω*_p_ in the telecom regime and their *ω*_p_ can even be tuned by controlling the doping level^68,69^. These materials enable ENZ-based light-matter interactions in various frequency regimes.

Distinct from the longitudinal wave supported by volume plasmons, DCZIMs can support the propagation of TM and/or TE waves. Using TM-polarized DCZIMs as an example, the electric field of the TM-polarized wave oscillates between an electric monopole mode and a transverse magnetic dipole mode, corresponding to zero effective permittivity (*ε*_*zz*_ = 0) and permeability (*μ*_*xx*_ = *μ*_*yy*_ = 0), respectively. Such an EMNZ behavior is essentially different from the ENZ behavior of volume plasmons. From the viewpoint of the impedance matching, EMNZ induces a finite intrinsic impedance $Z = \sqrt {\mu _{{{{\mathrm{eff}}}}}/\varepsilon _{{{{\mathrm{eff}}}}}}$ instead of the infinite impedance of ENZ. Consequently, EMNZ can be better impedance-matched with a regular medium, such as free space, optical fibers, and waveguides. From the viewpoint of losses, Dirac-like cone’s good coupling to free space brings large radiation losses, resulting in a short propagation length. A Dirac-like cone can provide a macroscopic refractive index of zero by averaging the electric and magnetic responses over the plane of the 2D photonic crystal. On the other hand, volume plasmons can provide a continuous zero refractive index over a bulk metal. The Dirac-like cone-based zero index can only replace the continuous zero refractive index for certain light-matter interactions, such as the normal incidence of transverse waves and the interactions of many dipoles which are randomly distributed throughout the zero-index medium.

EMNZ can be achieved by engineering a typical traditional metamaterial—metal-dielectric-metal fishnet structure^15^. Different from fishnet metamaterials, DCZIMs can be realized via purely dielectric structures, avoiding the ohmic losses. Fishnet structure can be simply fabricated in the out-of-plane configuration (light propagates perpendicular to the substrate) which can couple to free-space light efficiently, making it suitable for free-space-optical applications. On the other hand, the 2D-array structure of DCZIM can be easily fabricated in the in-plane configuration (light propagates parallel to the substrate) which can couple to integrated photonic waveguides efficiently, enabling applications in integrated photonics. Both DCZIM and fishnet metamaterial can be designed to show EMNZ behavior to arbitrary polarized incident light at normal incidence. And, both DCZIM and fishnet metamaterial are periodic structures consisting of subwavelength unit cells, showing macroscopic EMNZ behavior (“Homogenization of DCZIMs” section).

An arbitrarily shaped 2D ENZ medium doped with several 2D macroscopic nonmagnetic dielectric dopants is equivalent to a 2D homogeneous medium with the same shape, a near-zero effective permittivity, and an effective permeability determined by the geometric and material parameters of the dopants^16–19^. When the dopants’ parameters equal to certain values, the effective permeability equals to zero, leading to an EMNZ behavior near the plasma frequency of the ENZ host. The effective permittivity of the doped ENZ medium behaves the same as that of the ENZ host—a volume plasmon showing zero permittivity at the plasma frequency (Table 1). In the dispersion diagram, this EMNZ behavior corresponds to the dispersion curve crossing zero wavenumber at the plasma frequency, leading to a continuous change of the effective refractive index from a negative value at lower frequencies to a positive value at higher frequencies. This EMNZ phenomenon can be excited by a transverse electric (TE) polarized wave, enabling a transmission of such a wave through the doped ENZ medium without any spatial phase advance. Even the losses and dispersion of the ENZ host limit the performance of photonic doping, the desired zero permeability can still exist with moderate losses. And, because the EMNZ behavior corresponds to the dispersion curve in the radiation continuum above the light line, doped ENZ medium can couple to free space via transverse wave, resulting in radiation losses.

**Table 1:** Comparison of optical properties of volume plasmon, fishnet metamaterials, photonic doping, and DCZIMs

	Volume plasmon	Fishnet	Photonic doping^a^	DCZIM
*ε* and *μ*				
Dispersion diagram				
Wave property	*ω* ≤ *ω*_p_: longitudinal, *ω* > *ω*_p_: transverse	Transverse	Transverse	Transverse
Light-matter interaction	*ω* ≤ *ω*_p_: no interaction, *ω*>*ω*_p_: interaction	Interaction	Interaction	Couple with photon to form zero-index modes
Excitation	*ω* = *ω*_p_: uniform electric field, *ω*>*ω*_p_: transverse wave	Transverse wave	Transverse wave	Transverse wave
Propagation length	*ω* ≤ *ω*_p_: no propagation, *ω* > *ω*_p_: short	Short	Short	Short
Loss	Ohmic loss	Ohmic loss	Ohmic loss	Radiation loss
Homogeneity	Continuous zero index over a volume	Macroscopic zero index	Macroscopic zero index	Macroscopic zero index
References	^115^	^15^	^18^	^21^

^a^*ε*_eff_ of photonic doping as that of volume plasmon

Both doped ENZ media and DCZIMs show macroscopic EMNZ behavior—uniform field distribution outside the media and non-uniform field distribution inside the media. Distinct from the arbitrarily shaped ENZ media doped with dielectric dopants, DCZIMs consist of periodic structures, having less flexibility in forming an arbitrary-shaped geometry, especially when the local feature of the geometry is comparable to the size of the unit cells of the metamaterials. In contrast to the doped ENZ media which show EMNZ response to TE wave only, DCZIMs can show EMNZ response to both TE and/or TM waves. Because the ENZ hosts of doped ENZ media are usually made from metals, doped ENZ media always show a certain amount of ohmic losses, especially in the optical regime. On the other hand, DCZIMs can be made by purely dielectric structures, avoiding ohmic losses completely. Both DCZIMs and doped ENZ media correspond to dispersion curves in the radiation continuum above the light line, leading to radiation losses.

## Classification of DCZIMs

**Fig. 3:**
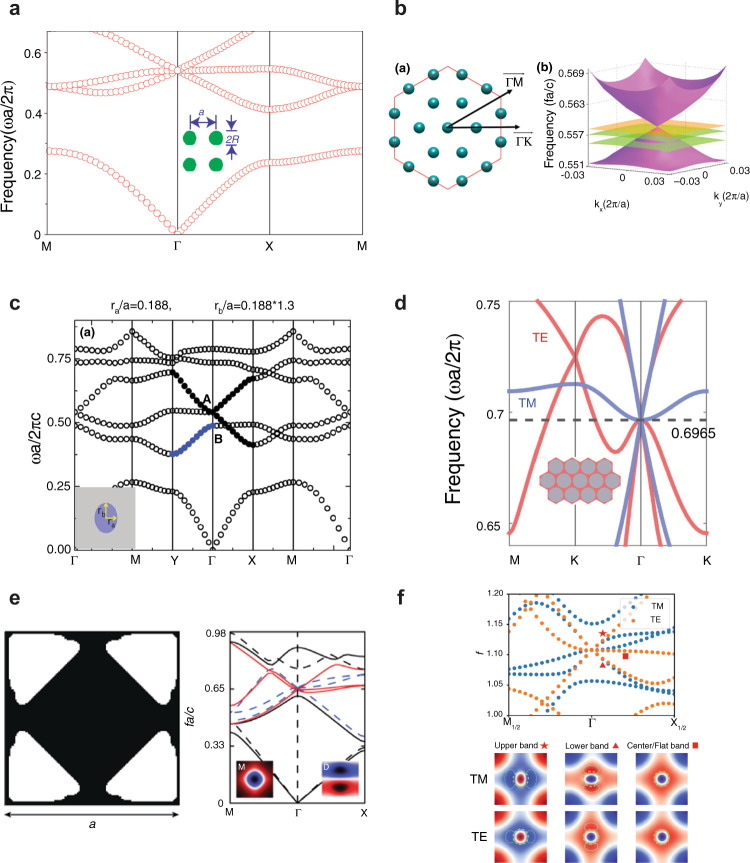
Photonic bandstructure and schematic of different types of DCZIMs. Dirac-like cones based on **a** a 2D photonic crystal with a square lattice of dielectric rods and **b** a 2D photonic quasicrystal of dielectric rods. **c** semi-Dirac-like cone based on a square lattice of elliptical dielectric rods. Full-polarization Dirac-like cones based on **d** a hyper-crystal consisting of dielectric rods in an elliptic metamaterial, **e** a photonic crystal designed by using topology optimization, **f** a square lattice of dielectric rods. Figure adapted with permission from: **a** ref. ^21^, Springer Nature Limited; **b** ref. ^70^, APS; **c** ref. ^71^, © The Optical Society; **d** ref. ^76^, Springer Nature Limited; **e** ref. ^52^, APS; **f** ref. ^54^, © The Optical Society

DCZIMs were originally realized by engineering the radius and lattice constant of 2D photonic crystals consisting of a square or triangular lattices of dielectric rods^21^ (Fig. 3a). These DCZIMs can show an impedance-matched and isotropic zero refractive index for TM-polarized light. Such a metamaterial can also be realized via a photonic quasicrystal of dielectric rods without translational symmetry (Fig. 3b)^70^. By changing the cross-section of the rods from a circle to an ellipse in a square lattice, an anisotropic semi-Dirac-like cone can be achieved with EMNZ in one direction and ENZ in the orthogonal direction (Fig. 3c)^71,72^. The semi-Dirac-like cone is useful for applications in directional emission^73^, asymmetric transmission^74^, beam deflection, beam splitting, and light focusing^75^. As most 2D DCZIMs are designed for either TM or TE polarization, full-polarization Dirac-like cones induced by the degeneracy of a TM Dirac-like cone and a TE Dirac-like cone at the same frequency can show zero-index behavior regardless of the polarization of the incident light. Full-polarization Dirac-like cones can be achieved via photonic hyper-crystals consisting of dielectric rods in an elliptic metamaterial (Fig. 3d)^76^, photonic crystals inversely designed by using topology optimization (Fig. 3e)^52^, a square lattice of dielectric veins^54^, or a square lattice of dielectric rods (Fig. 3f)^77^.

## Optical DCZIMs

**Fig. 4:**
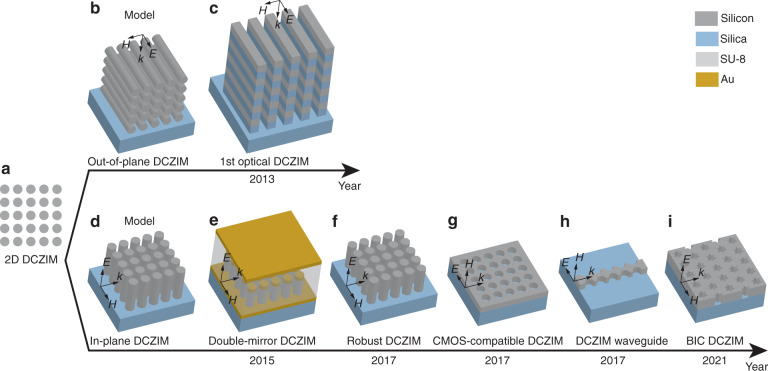
Development of optical DCZIMs. **a** Original theoretical design of DCZIM consists of a square array of 2D silicon pillars^21^. **b** Out-of-plane configuration of DCZIM with the plane of periodicity perpendicular to substrate. **c** First optical DCZIM realized in the out-of-plane configuration^56^. **d** In-plane configuration of DCZIM with the plane of periodicity parallel to substrate. **e** In-plane DCZIM consists of a square array of silicon pillars embedded within a SU-8 matrix and clad by gold mirrors^57^. **f** In-plane DCZIM shows robustness against the fabrication imperfection in the radii of pillars^79^. **g** In-plane CMOS-compatible DCZIM consists of a square array of air holes in a 220-nm silicon on insulator^51^. **h** In-plane 1D DCZIM waveguide^31^. **i** In-plane DCZIM achieves ultra-low propagation loss via bound states in the continuum (BIC)^53,55^

Based on the original theoretical design of DCZIM (Fig. 4a), most optical DCZIMs are implemented by using standard planar processes in two configurations: out-of-plane configuration in which light propagates in the direction perpendicular to the substrate (Fig. 4b), and in-plane configuration where light propagates in the direction parallel to the substrate (Fig. 4d). The out-of-plane configuration can couple to free-space light efficiently, making it suitable for free-space-optical applications. The in-plane configuration can be fabricated over a large area in arbitrary shapes and can efficiently couple to integrated photonic circuits, enabling various zero index-based devices for integrated photonics.

**Fig. 5:**
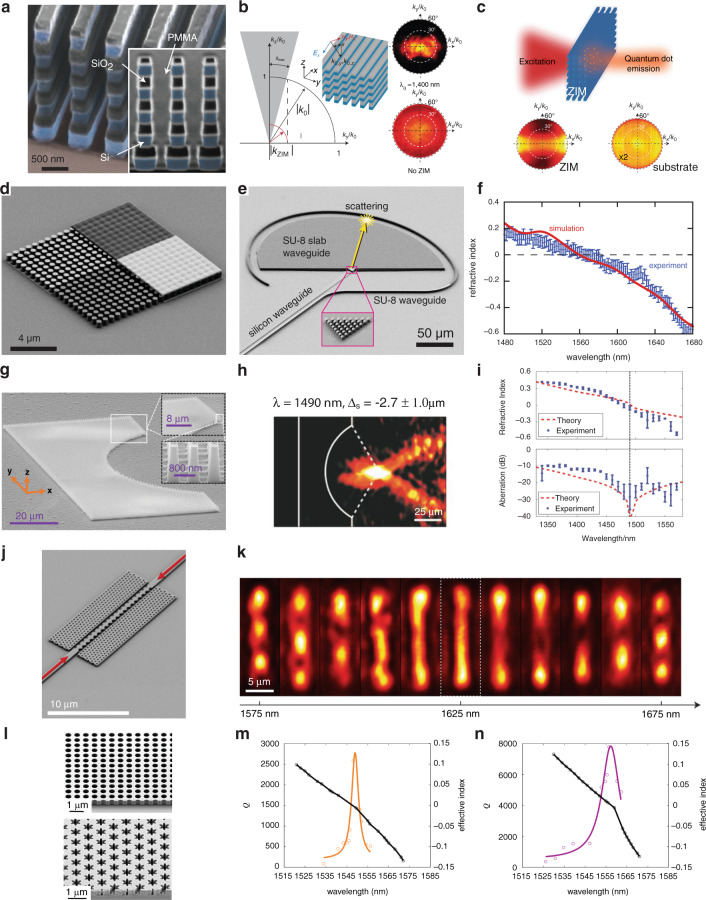
Optical DCZIMs’ structures, theoretical and experimental results. **a** Scanning electron microscopy (SEM) image of an out-of-plane zero-index metamaterial with the experimental demonstration of this metamaterial’s small angular selectivity **b** and directional emission from quantum dots within this metamaterial (**c**). **d** SEM images of an in-plane zero-index metamaterial and **e** the waveguides and metamaterial for measuring the zero index with **f** the simulated and measured effective indices. **g** In-plane zero-index lens with its optical microscope image (**h**), effective refractive index (top), and longitudinal spherical aberration (bottom) **i**. **j** SEM image of an in-plane zero-index waveguide with its measured interference patterns at different wavelengths (**k**). **l** SEM images of in-plane resonance-trapped (top) and symmetry-protected (bottom) BIC zero-index metamaterials with their measured quality factors and effective refractive indices, respectively (**m**, **n**). Figure adapted with permission from **a**–**c** ref. ^56^, Springer Nature Limited; **d**–**f** ref. ^57^, Springer Nature Limited; **g**–**i** ref. ^78^, American Chemical Society; **j**–**k** ref. ^31^, American Chemical Society; **l**–**n** ref. ^55^, American Chemical Society

The first optical DCZIM was realized in an out-of-plane configuration by patterning 11 alternating layers of amorphous silicon and silica, forming 5 layers of silicon rods in the out-of-plane direction (Figs. 4c and 5a)^56^. This metamaterial’s transmission measurement shows an impedance-matched low index at 1409 nm followed by a bandgap near 1460 nm, corresponding to a continuous spectrum with both *ε*_eff_ and *μ*_eff_ positive, *ε*_eff_ negative and *μ*_eff_ positive, and both *ε*_eff_ and *μ*_eff_ negative. This result is further confirmed by the measured transmission in a small angular regime of the Fourier plane at the low-index wavelength, which is due to the small critical angle at the interface between air and a low-index medium (Fig. 5b). To further verify the near-zero index of the metamaterial, emissions from the quantum dots, whose luminescence peak is around the near-zero-index wavelength, embedded in the metamaterial and an un-patterned PMMA film were measured. When compared with the un-patterned sample, the metamaterial significantly enhances both the angular confinement and the emission in the direction normal to the interface (Fig. 5c). The enhanced angular confinement is due to the small critical angle at the interface between air and the near-zero-index medium. The enhanced emission is caused by the constructive interference of the emitted light over the almost uniform spatial phase distribution throughout the metamaterial.

An in-plane DCZIM was achieved by sandwiching low-aspect-ratio silicon pillars between two gold films based on a silicon-on-insulator chip (Figs. 4e and 5d)^57^. According to imaging theory, the two gold films serve as mirrors to extend the optical height of the silicon pillars, realizing effective 2D silicon pillars with infinite height. To experimentally measure the zero index of this metamaterial, a prism consisting of 8 by 8 metamaterial unit cells was fabricated. Light is guided into this prism via a silicon tapered waveguide operating in the fundamental TM mode, and refracted into a semicircular SU-8 slab waveguide (Fig. 5e). The refraction angle is obtained by measuring the scattered light at the semicircular edge of the SU-8 slab waveguide. Measured results show that the effective refractive index of this metamaterial crosses zero linearly near 1570 nm, corresponding to a Dirac-like cone dispersion at the center of the Brillouin zone (Fig. 5f).

Another in-plane DCZIM was realized by fabricating high-aspect-ratio silicon pillars from a single crystalline silicon substrate^78^. This metamaterial was fabricated over a large area to form a planar concave lens with wavelength-scale thickness (Fig. 5g). As light transmitting through a zero-index medium is always refracted in the direction normal to the interface, the zero-index concave lens can focus all the refracted ray on the concave center (Fig. 5h), eliminating the longitudinal spherical aberration induced by the difference between the focal spots of paraxial and off-axial light. Such an extraordinary focusing effect was measured by probing the light scattered by the irregular silicon substrate. The effective refractive index of the metamaterial can be retrieved from the measured position of the focal point. Experimental results show that the effective index continuously changes from a positive value at short wavelengths to a negative value at longer wavelengths, indicating a zero crossing at 1490 nm (Fig. 5i, top). Results also show that the zero-index lens has a longitudinal spherical aberration of −28.3 ± 3.7 dB at the zero-index wavelength, which is at least 10 dB smaller than that at other wavelengths (Fig. 5i, bottom).

The impedance-matched zero index provided by a DCZIM is not robust against fabrication imperfections because the Dirac-like cone dispersion is formed by an accidental degeneracy that can only exist for a particular combination of geometric parameters. Even a small change in the geometric parameters can break the accidentally degenerated Dirac-like cone into a bandgap. To overcome this drawback, an in-plane all-dielectric DCZIM whose Dirac-like cone dispersion is robust against changes in the radius of pillars was demonstrated (Fig. 4f)^79^. Radius was chosen because its fabrication error is much larger than those associated with the lattice constant and the height of pillars. Considering that the Dirac-like cone is induced by the degeneracy of an electric monopole mode and a transverse magnetic dipole mode at the Γ point, the pitch and height were designed to guarantee that the Γ point wavelengths and equivalent indices, respectively, of monopole and dipole modes are equal to each other over a large range of radii. Experimental results show that zero-crossing of the effective refractive index redshifts from ~1560 to ~1620 nm as the radius increases from 229.5 to 251.5 nm, verifying the robustness of this design against changes in the pillar radius.

An in-plane, monolithic, CMOS-compatible DCZIM was achieved by fabricating a square lattice of air holes in a standard 220-nm-thick silicon-on-insulator (SOI) (Fig. 4g)^51^. This metamaterial can be patterned on a photonic chip in a single step together with other components of a photonic integrated circuit. The measured effective refractive index of this metamaterial linearly changes from 0.51 ± 0.04 at 1480 nm to −0.21 ± 0.05 at 1680 nm, crossing zero at 1625 nm. This metamaterial enables the mass production of zero-index-based devices at low cost and with high quality via CMOS fabrication techniques.

To reduce zero-index metamaterials’ cross-section in the transverse plane for applications in nonlinear optics and integrated photonics, one row of the CMOS-compatible DCZIM^51^ was taken to realize a zero-index waveguide (Figs. 4h and 5j)^31^. To measure the near-zero effective index of this waveguide, a standing wave is formed in the waveguide by illuminating it from both ends simultaneously. Because the distance between successive nodes or antinodes of a standing wave in a low-index medium is above the diffraction limit, this distance can be resolved in the far-field by measuring the out-of-plane radiation from the zero-index waveguide (Fig. 5k). The effective wavelength and effective refractive index of the waveguide can be extracted from the measured distance between successive antinodes of the standing wave, showing an effective refractive index of zero near 1627 nm.

Because the transverse dipole mode forming the Dirac-like cone is in the radiation continuum above the light line, in-plane DCZIMs have large radiation losses in both the in-plane and out-of-plane directions, resulting in a propagation loss as high as 1.12 dB μm^−1^^51^. Such a large out-of-plane radiation loss can be mitigated using the concept of bound states in the continuum (BIC)—photonic modes in the radiation continuum above the light line but are confined in the photonic crystal slab with an infinite quality factor^80,81^. BIC DCZIMs have been implemented in at least two ways: eliminating the out-of-plane radiation of the transverse dipole mode via resonance-trapped BIC which causes destructive interference in the far-field^9,82,83^; and, forming Dirac-like cone dispersion via symmetry-protected modes without out-of-plane radiation^53,55^. The resonance-trapped BIC zero-index metamaterial consists of a square lattice of air holes in 570-nm-thick SOI whereas the symmetry-protected BIC zero-index metamaterial consists of a hexagonal lattice of daisy-shaped air holes in 370-nm-thick SOI (Figs. 4i and 5l). Experimental results show that resonance-trapped and symmetry-protected BIC zero-index metamaterials have quality factors of 2.6 × 10^3^ and 7.8 × 10^3^, respectively, near the zero-index wavelengths at 1548.0 nm and 1558.0 nm (Fig. 5m, n). The symmetry-protected BIC zero-index metamaterial shows a propagation loss that is one-order of magnitude smaller than the previous design^51^.

BIC zero-index metamaterials have several limitations. The resonance-trapped BIC zero-index metamaterials only show a low propagation loss when the thickness of the photonic crystal slab equals to a certain value, which is due to the resonance-trapped BIC’s high sensitivity to the variation in the thickness of photonic crystal slab^9^. In contrast, the low propagation loss of symmetry-protected BIC zero-index metamaterials is robust against the variations in geometric parameters due to the fact that symmetry-protected BIC modes show high-quality factors because of their intrinsic mode symmetry^53,55^. Because BIC only show a high quality factor (a low out-of-plane radiation loss) at a certain wavelength, BIC zero-index metamaterials only show high quality factors (low out-of-plane radiation losses) near the zero-index wavelengths (Fig. 5m, n).

## Applications

**Fig. 6:**
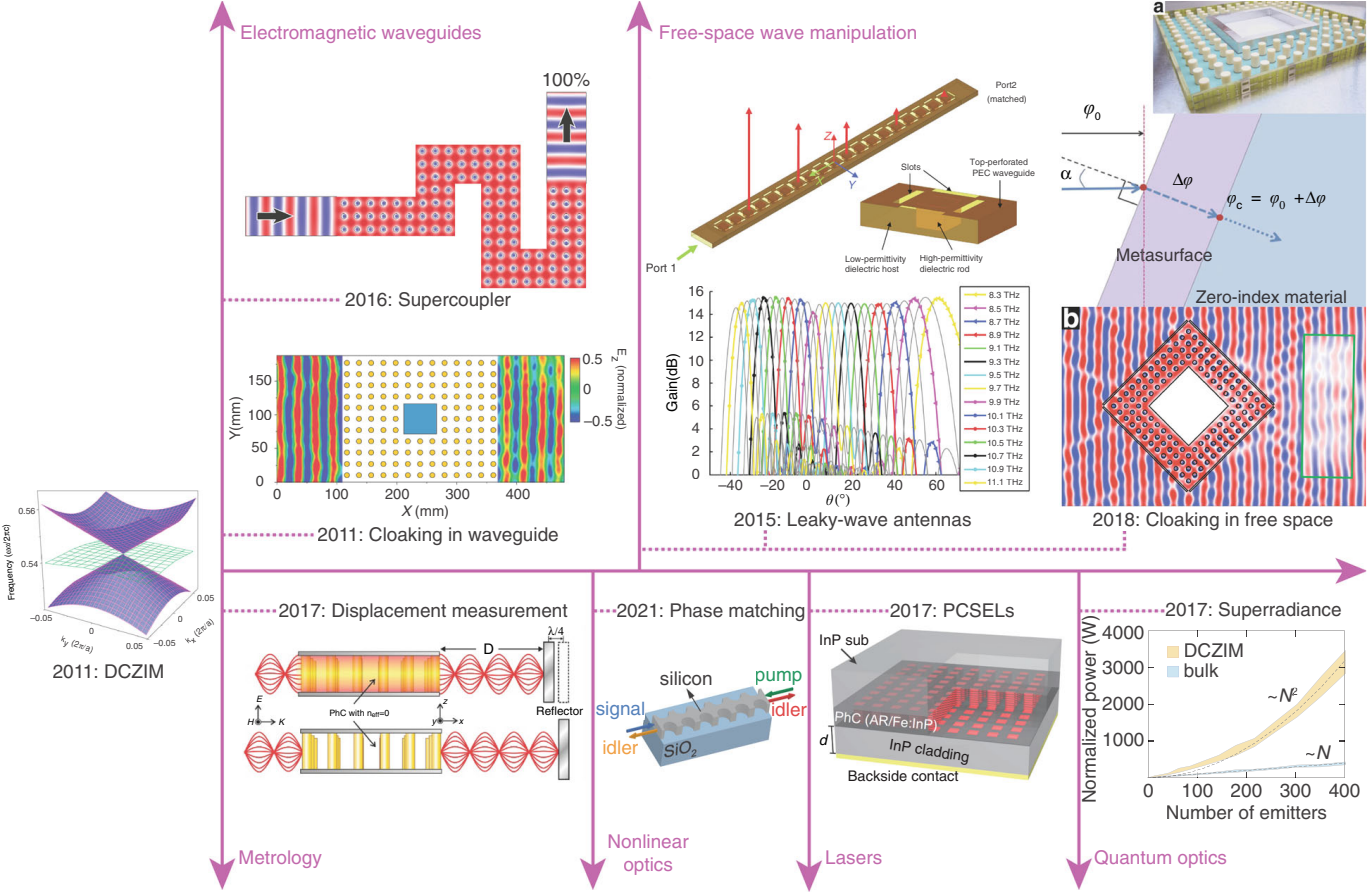
Selected applications of DCZIMs in (from left to right, top to bottom) electromagnetic waveguides, free-space wave manipulation, metrology, nonlinear optics, lasers, and quantum optics. Supercoupler: simulated electric field in a meandering waveguide filled with DCZIMs. Cloaking in waveguides: measured electric field outside a DCZIM embedded with a metallic object. Leaky-wave antennas: schematic of the leaky-wave antenna based on a DCZIM (top) and the simulated gain at different operating frequencies (bottom). Cloaking in free space: the fabricated cloak consisting of a metasurface and a DCZIM with a central metallic rhombus as the hidden object (top); schematic shows that the metasurface changes the phase of incident wave from *ϕ*_0_ to *ϕ*_c_ (middle); the measured (inside green box) and simulated (outside green box) electric field of the cloak area. Displacement measurement: schematic of the displacement measurement based on a DCZIM. Phase matching: Direction-independent phase matching of four-wave mixing in a DCZIM waveguide. PCSELs: a photonic-crystal surface-emitting laser (PCSEL) operating at a Dirac-point mode. Superradiance: simulated total power radiated from different-sized diamond DCZIMs embedded with randomly positioned dipole emitters. Figure adapted with permission from supercoupler^86^, PQDD; cloaking in waveguide^21^, Springer Nature Limited; leaky-wave antennas^88^, Springer Nature Limited; cloaking in free space^90^, Springer Nature Limited; displacement measurement^91^, © The Optical Society

By fully leveraging DCZIMs’ unique electromagnetic property—the infinite effective spatial wavelength, promising potential applications of DCZIMs have been demonstrated in electromagnetic waveguides, free-space wave manipulation, metrology, nonlinear optics, lasers and quantum optics (Fig. 6). In this section, we review DCZIMs’ applications whose performances are or may go beyond the state of the arts. For example, compared with the state-of-the-art direction-independent phase matching in a fishnet metamaterial over an interaction length (800 nm) shorter than the free-space wavelength (1510 nm)^7^, DCZIM waveguide achieved, at similar wavelengths, the direction-independent phase matching over an interaction length almost 10 free-space wavelengths (14.8 μm)^84^.

### Electromagnetic waveguides

For a TM-polarized (TE-polarized) transverse wave propagating within a 2D zero-index medium, the electric (magnetic) field polarized along the axis of the 2D zero-index medium does not vary as a function of space, resulting in a “quasi-spatial static field” at optical frequencies. This property can be used to achieve the effects of super-coupling and cloaking in waveguide. Super-coupling is a phenomenon that a continuous wave can tunnel through a narrow channel filled with ENZ medium with high transmission regardless of the shape, bends, and twists of the channel^3^. From the viewpoint of metallic waveguides, super-coupling is an effect that the single guided mode supported by a metallic waveguide filled with a zero-index medium is independent of the waveguide geometry. By taking advantage of this flexibility, a waveguide filled with a near-zero-index medium can be designed for resonant tunneling even when the waveguide geometry varies in a large range^85^. According to this concept, waveguides filled with DCZIMs have been designed (Fig. 6: supercoupler)^86^. The waveguide filled with a DCZIM can also make an embedded object invisible, in which light can perfectly transmit through the DCZIM embedded with any object (Fig. 6: cloaking in waveguide). On the other hand, the waveguide filled with a DCZIM can achieve coherent perfect absorption by doping the DCZIM with absorptive defects^87^.

### Free-space wave manipulation

By taking advantage of the out-of-plane radiation of the in-plane DCZIMs, we can achieve leaky-wave antennas for continuous beam scanning through broadside by varying the operating frequency^88^. Such continuous beam scanning is enabled by the linear dispersion of the gapless Dirac-like cone in the radiation continuum above the light line, corresponding to the continuity of the direction of the beam’s wavevector across the broadside. The leaky-wave antennas can be implemented in the microwave and terahertz regimes as dielectric-filled, parallel-plate waveguides with the top perfect-electric-conductor plate perforated to allow out-of-plane radiation out of the waveguide (Fig. 6: leaky-wave antennas, top). Simulation results show that in this design, the beam can be steered from 61° to −37° as the frequency is varied from 8.3 to 11.12 THz with a gain variation less than 2 dB (Fig. 6: leaky-wave antennas, bottom). To avoid metal’s ohmic losses in the optical regime, another leaky-wave antenna is designed in the form of in-plane DCZIMs in the telecom regime. The beam in this design can be steered from 86° to 93° as the frequency is varied from 198 to 204 THz.

Because the cloaking effect of waveguide filled with a DCZIM is induced by resonant tunneling instead of transformation optics^89^, scattering can be eliminated for normal incident wave only. Such a limitation can be overcome by coating the DCZIM with a transparent metasurface (Fig. 6: cloaking in free space, top), which redirects the wavefront of the incident wave to the direction normal to the surface of the metamaterial (Fig. 6: cloaking in free space, middle)^90^. This hybrid cloak was experimentally realized in the microwave regime and showed a good cloaking effect (Fig. 6: cloaking in free space, bottom), featuring an almost arbitrary shape. Moreover, this hybrid cloak can achieve a subwavelength thickness by replacing DCZIMs with other thin zero-index materials such as volume plasmon and fishnet metamaterials.

### Metrology

DCZIMs can be used in measuring subwavelength displacement^91^. As shown in Fig. 6: displacement measurement, by sending a continuous wave to a reflector, a standing wave can be formed via the interference between the incident and reflected waves. To measure a quarter free-space wavelength displacement of the reflector, we need to measure the spatial distance between a node and its neighboring antinode, which equals to a quarter free-space wavelength and is smaller than the diffraction limit. To measure this spatial distance in free space, we have to use super-resolution imaging techniques. If a zero-index metamaterial is placed where the standing wave’s antinode interfaces with the metamaterial’s input facet (right-hand-side facet in Fig. 6: displacement measurement, top), the amplitude of the uniform field would be maximized throughout the metamaterial. If the reflector is then moved by a distance equivalent to a quarter of the free-space wavelength, the node of the standing wave will interface with the metamaterial’s input facet (right-hand-side facet in Fig. 6: displacement measurement, bottom), minimizing the amplitude of the uniform field throughout the metamaterial. Hence, we can measure the displacement of the reflector by observing the intensity of the field within the metamaterial, whose size can be much larger than the diffraction limit. Such a displacement measurement method can provide a resolution better than a quarter of the free-space wavelength without using super-resolution imaging techniques. Experimental results show that the contrast of the measured field intensity is as large as ~55%, corresponding to a displacement of ~5 mm, which is approximately a quarter of the operating wavelength, 20.7 mm.

### Nonlinear optics

In general, DCZIMs can be used in nonlinear optics for phase matching and nonlinear enhancement. To generate a nonlinear signal efficiently over a long light-matter interaction length, the phase-matching condition Δ*k* = 0 must be met^92^. For four-wave mixing in zero-index metamaterials, the phase-matching condition Δ*k* = 2*γP*_pump_ − Δ*k*_L_ can be satisfied when the pump, signal, and idler are all in the near-zero-index regime near the Dirac-point frequency, in which the phase mismatch due to linear dispersion Δ*k*_L_ = 2*k*_pump_ − *k*_signal_ − *k*_idler_ compensates for the phase mismatch due to self-phase modulation and cross-phase modulation 2*γP*_pump_^7,84,93^. Furthermore, the isotropic zero index provided by a Dirac-like cone can satisfy the phase-matching condition in all directions in the plane of the array, enabling efficient direction-independent generation of nonlinear signals (Fig. 6: phase matching)^84^. Moreover, the out-of-plane radiation of on-chip metamaterials even enables excitation from the out-of-plane direction^84^. The finite impedance *Z* (e.g., 1.47^57^) corresponding to the zero index of DCZIMs can increase the nonlinear refractive index *n*_2_ = 3‧*Z*‧Re(*χ*^(3)^)/8^94^, boosting the nonlinear conversion efficiency. Such a nonlinear enhancement effect is similar to that provided by ENZ media, whose infinite impedance induces a larger nonlinear enhancement despite an impedance mismatch between the ENZ and a regular medium, such as air^95–97^.

For second-harmonic generation in zero-index metamaterials, we can satisfy the phase-matching condition Δ*k* = 2*k*_pump_ − *k*_signal_ = 0 by designing a DCZIM with a Dirac-like cone dispersion at the pump frequency and a bandedge at the second-harmonic frequency^98^.

Based on the strong field localization and enhancement within a DCZIM under a small incident angle^99^, we can achieve all-optical switching with a low threshold intensity^100^.

### Lasers

Lasing modes of conventional photonic crystal surface-emitting lasers (PCSELs) usually show quadratic dispersion in the photonic bandstructure. Such PCSELs’ lasing areas are limited by two fundamental factors: a larger cavity area induces smaller mode spacings, resulting in multi-mode lasing; and, the in-plane feedback divides the lasing fields into individual coherent sections, leading to multi-area lasing. By using a linear-dispersion mode of the Dirac-like cone as the lasing mode, we can dramatically increase the mode spacings and eliminate the in-plane feedback, enabling larger-area single-mode PCSELs with higher output power^101^. To ensure that a linear-dispersion mode of the Dirac-like cone is selected for lasing, DCZIM consisting of a square lattice of pillars made from semiconductor heterostructures is designed, providing a feasible approach to realize electrically pumped Dirac-like cone-based PCSELs (Fig. 6: PCSELs)^102^.

### Quantum optics

DCZIMs can be used in quantum optics to engineer emission from quantum emitters. A unidirectional single-photon source with almost 100% efficiency can be achieved by placing a quantum emitter inside a Fabry–Perot cavity whose mirrors are made of negative-index and zero-index metamaterials^103^. The inner negative-index slabs function as a cage to trap the photon within the cavity, while the outer zero-index slabs work as a filter allowing transmission at normal incidence only, thereby enabling unidirectional generation of single photon. The negative-index metamaterial consists of a hexagonal lattice of air holes in a dielectric substrate^104^ while the zero-index metamaterial consists of a square lattice of dielectric pillars^21^. Simulation results confirmed the unidirectional single-photon generation at 1.55 μm.

Super-radiance is a many-body phenomenon in which emitters radiate coherently, and the constructive interference leads to an *N*-fold increase in the spontaneous emission rate, where *N* is the number of emitters^105,106^. To satisfy the phase-matching condition for perfect coherence, super-radiance requires all the emitters within one wavelength from one another, limiting super-radiance to a small number of emitters and over a small spatial extent. These limitations could be overcome by taking advantage of the infinite spatial wavelength and perfect spatial coherence of zero-index metamaterials. In one work, simulation results of multiple emitters in a homogenous near-zero-index medium show that the emission intensity is proportional to the number of emitters squared, a hallmark of super-radiance^56^. In another work, analytical and numerical results demonstrate the enhanced super-radiant emission of silicon vacancy centers (SiVs) in a diamond DCZIM whose size is far beyond the emission wavelength near 737 nm^8^. This diamond DCZIM consists of a square array of 1-μm tall diamond pillars on a diamond substrate for TM polarization. SiVs are placed near the center of each diamond pillar to only excite and couple to the zero-index monopole mode, which lets all the SiVs experience a relative uniform field distribution (“Relationship between a Dirac-cone dispersion...” section). As a result, the total radiated power increases in proportion to the square of the number of SiVs, verifying the super-radiant phenomenon (Fig. 6: superradiance). By contrast, the total radiated power from SiVs in unstructured (bulk) diamond increases linearly with the number of SiVs. This is consistent with incoherent emission, due to the random positions of the SiVs.

## Summary and outlook

When compared with other mechanisms including volume plasmons, fishnet metamaterials, doped ENZ media for realizing a near-zero refractive index, DCZIMs which are essentially dielectric photonic crystals have the unique feature of using only dielectric structures. Such a feature enables fabricating photonic DCZIMs in the form of zero-index waveguiding structures using standard planar processes in SOI wafers. These zero-index waveguiding structures facilitate the interactions between light and zero-index medium over a large area in arbitrary shapes on a photonic chip. Based on these zero-index waveguiding structures, the interaction length of phase mismatching-free nonlinear signal generation can be increased from sub-free-space wavelength scale (fishnet metamaterials)^7^ to almost 10 free-space wavelengths^84^, integrated zero-index waveguides with arbitrary shapes may be realized, larger-area single-mode PCSELs with higher output power might be achieved^101^, extended superradiance may be realized for many emitters over a large spatial extent^8^. Hence, DCZIMs can serve as a scalable and flexible on-chip platform for exploring the rich physics and potential applications of zero index.

On the other hand, DCZIMs also have some limitations when compared with other mechanisms for realizing a near-zero refractive index. The macroscopic zero index provided by DCZIMs can only replace the continuous zero index provided by volume plasmons for certain light-matter interactions. And, in contrast to the fishnet metamaterials, which can be simply fabricated in the out-of-plane configuration and hence can couple to free-space light efficiently for free-space-optical applications^15,65^, it is more challenging to fabricate DCZIMs in the out-of-plane configuration for applications in free-space optics^56^. Moreover, different from the arbitrarily shaped-doped ENZ media^18^, DCZIMs consist of periodic structures, limiting its flexibility in forming an arbitrarily shaped geometry, especially when the local feature of the geometry is comparable to the size of the unit cell. Hence, we should choose the mechanism to achieve a near-zero refractive index according to the particular application.

So far, most potential applications of DCZIMs are only predicted by theoretical results while a few are demonstrated through proof-of-concept experiments. To further develop those potential applications and even achieve performance beyond the state of the arts, such as zero-index waveguides whose overall performance is better than that of silicon waveguides, we envision “customized” DCZIMs designed to satisfy the particular requirements of certain applications. In the following sections, we try to provide the design methodology of DCZIMs for their applications in optical interconnects, nonlinear optics, lasers, and quantum optics.

### Optical interconnects

The most intriguing application of DCZIMs in linear optics may be super-couplers whose minimum bending radius could be smaller than that of silicon waveguides (around 5 μm at 1500 nm^107^). This feature could increase the density of photonic integration of optical interconnects significantly. However, a super-coupler is made by filling a metallic waveguide with a zero-index medium, hampering the realization of optical super-couplers due to the high losses of metals in the optical regime. To achieve super-couplers with low propagation losses at optical frequencies, we envision zero-index waveguides consisting of a linear array of unit cells of the DCZIMs—a periodic dielectric waveguide showing a Dirac-cone dispersion at *k* = 0. Such a zero-index waveguide’s radiation losses could be eliminated by introducing a BIC in both the out-of-plane and in-plane directions^80^ or by inversely designing Dirac-like cone dispersion with modes without out-of-plane and in-plane radiations^108^. This zero-index waveguide’s bending losses could be decreased via structures with the connected dielectric region along the propagation direction, such as fishbone^31^.

### Nonlinear optics

Because only a Dirac-cone dispersion at a long wavelength can rigorously correspond to the EMNZ behavior (“Relationship between a Dirac-cone dispersion...” section), it may unrealistic to achieve zero index-based phase-matching for nonlinear processes whose interacting waves’ frequencies are further away from each other, such as second-harmonic generation and third-harmonic generation. For second-harmonic generation, to satisfy the phase-matching condition Δ*k* = 2*k*_1_ − *k*_2_ = 0, the zero index-based phase matching requires the metamaterial showing zero-index behaviors at both the fundamental frequency *ω*_1_ and the second-harmonic frequency *ω*_2_. Even we can design a metamaterial with two Dirac-cone dispersions at *ω*_1_ and *ω*_2_, respectively, the metamaterial won’t behave as a bulk homogeneous medium with an effective refractive index of zero at *ω*_2_ because the size of unit cell *a* is comparable to the free-space wavelength at *ω*_2_ (for a metamaterial consisting of a square array of 2D silicon pillars, *a*/*λ*_0_ ≈ 0.55 at *ω*_1_ and *a*/*λ*_0_ ≈ 1.1 at *ω*_2_).

Based on DCZIMs, we can realize zero index-based phase-matching for nonlinear processes whose interacting waves’ frequencies are closing to each other, such a four-wave mixing^84,93^. To improve the nonlinear conversion efficiency, we shall design a zero-index waveguide with a low propagation loss and a small effective mode area. To increase the frequency range of the interacting waves, we could design a zero-index waveguide with a low-loss near-zero index over a relatively large bandwidth. However, DCZIMs are always dispersive and can only show a near-zero index in the vicinity of the Dirac-point frequency, but the bandwidth may be improved by designing the metamaterial to approach the ENZ bandwidth of the Drude model—the widest bandwidth of a near-zero index of passive low-loss media.

### Lasers

Further development of Dirac-like cone-based PCSELs is limited by the following constraints: the index contrast between the pattern and semiconductor cladding is too low to design a DCZIM, and the quality factors of Dirac-like cone modes are too close to each other to provide enough mode selection for single-mode lasing^109,110^. The first constraints might be overcome by carefully selecting materials for the pattern and cladding. We may overcome the second constraint by engineering quality factors of all the modes near the Dirac-cone wavelength using inverse design^52^.

### Quantum optics

Although extended superradiance could be realized in a DCZIM with a large number of emitters over a large spatial extent^8^, it may be better to achieve superradiance in a purely dielectric ENZ metamaterial. Such an ENZ metamaterial could be realized by breaking the accidental degeneracy of modes forming the Dirac-cone dispersion (“Relationship between a Dirac-cone dispersion....” section), resulting in a monopole mode at the center of the Brillouin at a long wavelength. Such a monopole mode could show ENZ behavior. The advantages of this ENZ metamaterial over DCZIM includes: first, the huge impedance mismatch between the ENZ metamaterial and the ambient media confines most of the light within the metamaterial; second, the large impedance of ENZ metamaterial corresponds to a slow-light effect, leading to an enhancement in the spontaneous emission^111,112^. Furthermore, this purely dielectric ENZ metamaterial is also advantageous over metallic ENZ systems such as plasmonic channels^113^ and ENZ nanoscale waveguides^114^ due to its low propagation losses and arbitrary shapes over the plane of the pattern.

## Supplementary information

Reference_8_Mello2017

Reference_85_Gagnon2021

Reference94_Reshef2016

Reference103_Liang2017

Permission_Figure3a_Figure6-2011DCZIM_2011CloakingWaveguide

Permission_Figure3b

Permission_Figure3c_Figure6-2017Displacement_measurement

Permission_Figure3d

Permission_Figure3e

Permission_Figure3f

Permission_Figure5abc

Permission_Figure5ghi

Permission_Figure5def

Permission_Figure5jk

Permission_Figure5lmn

Permission_Figure6-2015Leaky_wave_antennas

Permission_Figure6-2016Supercoupler

Permission_Figure6-2017PCSELs

Permission_Figure6-2017Superradiance

Permission_Figure6-2018Cloaking_in_free_space

## Data Availability

The data that support the original figures within this review is available from the corresponding author upon reasonable request.

## Code availability

The code used in this review is available from the corresponding author upon reasonable request.
