# Supplementary material for: Dirac-like cone-based electromagnetic zero-index metamaterials
Source: Light Sci Appl. 2021 Sep 30;10:203. doi: 10.1038/s41377-021-00642-2 (PMC8481486; doi:10.1038/s41377-021-00642-2)
Supplement: Supplementary file 3 — Reference94_Reshef2016 [file 41377_2021_642_MOESM3_ESM.pdf]

Subject:	RE: Request permission of reusing two figure in two papers published in Optics Express
From:	pubscopyright <copyright@osa.org> Aug 17, 2021 9:20:41 PM
To:	"李杨" <yli9003@mail.tsinghua.edu.cn>, pubscopyright <copyright@osa.org>

Dear Yang,

Thank you for contacting The Optical Society.

For the use of figure 1 from Ying Wu, "A semi-Dirac point and an electromagnetic topological transition in a dielectric photonic crystal," Opt. Express 22, 1906-1917 (2014):

> OSA considers your requested use of its copyrighted material to be Fair Use under United States Copyright Law. It is requested that a complete citation of the original material be included in any publication.

For the use of figure 2 from Guoyan Dong, Zhenfei Li, Ji Zhou, Pengwu Qiao, Xiulun Yang, and Xiangfeng Meng, "Precise displacement measurement in single-beam interferometry employing photonic metamaterial with effective zero-index," Opt. Express 25, 31509-31515 (2017):

> OSA considers your requested use of its copyrighted material to be Fair Use under United States Copyright Law. It is requested that a complete citation of the original material be included in any publication.

> As this article is published under the terms of the OSA Open Access Publishing Agreement, when adapting or otherwise creating a derivative version of an article published under OSA's OAPA, users must maintain attribution to the author(s) and the published article's title, journal citation, and DOI. Users should also indicate if changes were made and avoid any implication that the author or OSA endorses the use.

While your publisher should be able to provide additional guidance, OSA prefers the below citation formats:

> For citations in figure captions:

> [Reprinted/Adapted] with permission from [ref #] © The Optical Society. (Please include the full citation in your reference list)

> For images without captions:

> Journal Vol. #, first page (year published) An example: Biomed. Opt. Express 6, 793 (2015)

Please let me know if you have any questions.

Kind Regards,

Hannah Greenwood

Hannah Greenwood

August 17, 2021

Authorized Agent, The Optical Society

The Optical Society (OSA)

2010 Massachusetts Ave., NW

Washington, DC 20036 USA

www.osa.org

Reflecting a Century of Innovation

From: 李杨 <yli9003@mail.tsinghua.edu.cn>
Sent: Friday, August 13, 2021 11:22 PM
To: pubscopyright <copyright@osa.org>
Subject: Request permission of reusing two figure in two papers published in Optics Express

Dear OSA Editors:

My name is Yang Li, I am a faculty member of the department precision instrument at Tsinghua University, Beijing. At this moment, I plan to reuse following figures in following two papers in our coming review paper with the title "Dirac-like cone-based electromagnetic zero-index metamaterials" which is recently accepted by the journal "Light: Science and Applications".

1. Figure 1a:

Y. Wu, "A semi-Dirac point and an electromagnetic topological transition in a dielectric photonic crystal," Optics Express, vol. 22, no. 2, pp. 1906-1917, 2014.

2. Figure 2b:

G. Dong, Z. Li, J. Zhou, P. Qiao, X. Yang, and X. J. O. e. Meng, "Precise displacement measurement in single-beam interferometry employing photonic metamaterial with effective zero-index," Optics Express, vol. 25, no. 25, pp. 31509-31515, 2017.

Could you please give me permission to reuse these figures in our review paper? Thank you!

Best regards,

Yang

--

李杨

副教授

清华大学，精密仪器系

通讯地址：北京市，海淀区，清华大学，9003大楼301-1

手机号：16601021689

电子邮箱：yli9003@mail.tsinghua.edu.cn; 20002000.leon@gmail.com

网页：http://faculty.dpi.tsinghua.edu.cn/yli9003.html (系个人主页)

http://yligroup.com/ （课题组）

Yang Li

Associate Professor

The Department of Precision Instrument, Tsinghua University

Yang Li

Room 301-1, 9003 Building

Tsinghua University

Haidian District

Beijing, China 100084

Tel: +86.16601021689 (Mobile)

E-mail: yli9003@mail.tsinghua.edu.cn; 20002000.leon@gmail.com

Website: http://faculty.dpi.tsinghua.edu.cn/en_yli9003.html (department profile)

http://yligroup.com/ (group)
