# Supplementary material for: Dirac-like cone-based electromagnetic zero-index metamaterials
Source: Light Sci Appl. 2021 Sep 30;10:203. doi: 10.1038/s41377-021-00642-2 (PMC8481486; doi:10.1038/s41377-021-00642-2)
Supplement: Supplementary file 4 — Reference103_Liang2017 [file 41377_2021_642_MOESM4_ESM.pdf]

Help Email Support

Full Polarization Conical Dispersion and Zero-Refractive-Index in Two-Dimensional Photonic Hypercrystals

Author: Jia-Rong Wang et al

Publication: Scientific Reports

Publisher: Springer Nature

Date: Mar 9, 2016

Copyright © 2016, The Author(s)

Creative Commons

This is an open access article distributed under the terms of the Creative Commons CC BY license, which permits unrestricted use, distribution, and reproduction in any medium, provided the original work is properly cited.

You are not required to obtain permission to reuse this article.

To request permission for a type of use not listed, please contact Springer Nature

© 2021 Copyright - All Rights Reserved | Copyright Clearance Center, Inc. | Privacy statement | Terms and Conditions
Comments? We would like to hear from you. E-mail us at customercare@copyright.com
