# Supplementary material for: Dirac-like cone-based electromagnetic zero-index metamaterials
Source: Light Sci Appl. 2021 Sep 30;10:203. doi: 10.1038/s41377-021-00642-2 (PMC8481486; doi:10.1038/s41377-021-00642-2)
Supplement: Supplementary file 9 — Permission_Figure3e [file 41377_2021_642_MOESM9_ESM.pdf]

Subject:	Thank you for your order with RightsLink / Springer Nature
From:	no-reply@copyright.com Apr 7, 2021 8:31:11 PM
To:	yli9003@mail.tsinghua.edu.cn

SPRINGER NATURE

Thank you for your order!

Dear Yang Li,

Thank you for placing your order through Copyright Clearance Center’s RightsLink® service.

Order Summary

Licensee:	Tsinghua University
Order Date:	Apr 7, 2021
Order Number:	5043630212710
Publication:	Nature Photonics
Title:	On-chip zero-index metamaterials
Type of Use:	Journal/Magazine
Order Total:	0.00 USD

View or print complete details of your order and the publisher's terms and conditions.

Sincerely,

Copyright Clearance Center

Tel: +1-855-239-3415 / +1-978-646-2777
customercare@copyright.com
https://myaccount.copyright.com

Copyright Clearance Center RightsLink®

This message (including attachments) is confidential, unless marked otherwise. It is intended for the addressee(s) only. If you are not an intended recipient, please delete it without further distribution and reply to the sender that you have received the message in error.
