# Supplementary material for: Dirac-like cone-based electromagnetic zero-index metamaterials
Source: Light Sci Appl. 2021 Sep 30;10:203. doi: 10.1038/s41377-021-00642-2 (PMC8481486; doi:10.1038/s41377-021-00642-2)
Supplement: Supplementary file 13 — Permission_Figure5def [file 41377_2021_642_MOESM13_ESM.pdf]

Subject:	Re: Request permission of using a figure of your PhD thesis
From:	"Sarah Camayd-Muñoz" <camayd.munoz@gmail.com> Aug 14, 2021 9:58:42 AM
To:	"李杨" <yli9003@mail.tsinghua.edu.cn>

Hi Yang,

Yes, please go ahead.

Best,
Sarah

On Fri, Aug 13, 2021 at 11:02 AM 李杨 <yli9003@mail.tsinghua.edu.cn> wrote:

> Hello Sarah:
>
> Please see figure 3.9 in the attachment.
>
> Best regards,
>
> Yang
>
> > -----Original Messages-----
> > **From:**"李杨" <yli9003@mail.tsinghua.edu.cn>
> > **Sent Time:**2021-08-12 01:54:47 (Thursday)
> > **To:** "Sarah Camayd-Munoz" <camayd.munoz@gmail.com>
> > **Cc:**
> > **Subject:** Request permission of using a figure of your PhD thesis
> >
> > Hello Sarah:
> >
> > Currently, I am preparing a review article on the topic of "Dirac-like cone-based electromagnetic zero-index metamaterials" and would like to reuse Figure 3.9 of your PhD thesis. At the position of reusing, I will cite your thesis properly. Could you please give me the permission to reuse this figure? Thank you!
> >
> > Best regards,
> >
> > Yang
> >
> > --
> > 李杨
> > 副教授
> > 清华大学，精密仪器系
> >
> > 通讯地址：北京市，海淀区，清华大学，9003大楼301-1
> > 手机号：16601021689
> > 电子邮箱：yli9003@mail.tsinghua.edu.cn; 20002000.leon@gmail.com
> > 网页：http://faculty.dpi.tsinghua.edu.cn/yli9003.html (系个人主页)
> > http://yligroup.com/ （课题组）
> >
> > Yang Li
> > Associate Professor
> > The Department of Precision Instrument, Tsinghua University
> >
> > Yang Li
> > Room 301-1, 9003 Building
> > Tsinghua University
> > Haidian District
> > Beijing, China 100084
> > Tel: +86.16601021689 (Mobile)
> > E-mail: yli9003@mail.tsinghua.edu.cn; 20002000.leon@gmail.com
> > Website: http://faculty.dpi.tsinghua.edu.cn/en_yli9003.html (department profile)
> > http://yligroup.com/ (group)
>
> --
> 李杨
> 副教授
> 清华大学，精密仪器系

通讯地址：北京市，海淀区，清华大学，9003大楼301-1
手机号：16601021689
电子邮箱：yli9003@mail.tsinghua.edu.cn; 20002000.leon@gmail.com
网页：http://faculty.dpi.tsinghua.edu.cn/yli9003.html (系个人主页)
http://yligroup.com/ （课题组）

Yang Li
Associate Professor
The Department of Precision Instrument, Tsinghua University

Yang Li
Room 301-1, 9003 Building
Tsinghua University
Haidian District
Beijing, China 100084
Tel: +86.16601021689 (Mobile)
E-mail: yli9003@mail.tsinghua.edu.cn; 20002000.leon@gmail.com
Website: http://faculty.dpi.tsinghua.edu.cn/en_yli9003.html (department profile)
http://yligroup.com/ (group)
