# Supplementary material for: Dirac-like cone-based electromagnetic zero-index metamaterials
Source: Light Sci Appl. 2021 Sep 30;10:203. doi: 10.1038/s41377-021-00642-2 (PMC8481486; doi:10.1038/s41377-021-00642-2)
Supplement: Supplementary file 15 — Permission_Figure5lmn [file 41377_2021_642_MOESM15_ESM.pdf]

Subject:	Re: Request permission of using a figure of our CLEO proceeding	
From:	"Mello, Olivia" <oliviamello@g.harvard.edu>	Aug 13, 2021 8:34:44 AM
To:	"李杨" <yli9003@mail.tsinghua.edu.cn>	

Absolutely! Thanks!

On Thu, Aug 12, 2021 at 2:21 AM 李杨 <yli9003@mail.tsinghua.edu.cn> wrote:

Olivia:

Currently, I am finalizing a review article on the topic of "Dirac-like cone-based electromagnetic zero-index metamaterials" and would like to reuse Figure 2c of our following CLEO proceeding:

O. L. Mello et al., "Strongly Extended Superradiance in Diamond Metamaterials," in Conference on Lasers and Electro-Optics, San Jose, California, 2017, p. JTu5A.26: Optical Society of America.

At the position of this reusing, I will cite our CLEO proceeding properly. Could you please give me the permission to reuse this figure? Thank you!

Best regards,

Yang

--
李杨
副教授
清华大学，精密仪器系

通讯地址：北京市，海淀区，清华大学，9003大楼301-1
手机号：16601021689
电子邮箱：yli9003@mail.tsinghua.edu.cn; 20002000.leon@gmail.com
网页：http://faculty.dpi.tsinghua.edu.cn/yli9003.html (系个人主页)
http://yligroup.com/ （课题组）

Yang Li
Associate Professor
The Department of Precision Instrument, Tsinghua University

Yang Li
Room 301-1, 9003 Building
Tsinghua University
Haidian District
Beijing, China 100084
Tel: +86.16601021689 (Mobile)
E-mail: yli9003@mail.tsinghua.edu.cn; 20002000.leon@gmail.com
Website: http://faculty.dpi.tsinghua.edu.cn/en_yli9003.html (department profile)
http://yligroup.com/ (group)

--
Olivia Mello
Department of Applied Physics
Harvard University
