# Supplementary material for: Dirac-like cone-based electromagnetic zero-index metamaterials
Source: Light Sci Appl. 2021 Sep 30;10:203. doi: 10.1038/s41377-021-00642-2 (PMC8481486; doi:10.1038/s41377-021-00642-2)
Supplement: Supplementary file 16 — Permission_Figure6-2015Leaky_wave_antennas [file 41377_2021_642_MOESM16_ESM.pdf]

SPRINGER NATURE

A hybrid invisibility cloak based on integration of transparent metasurfaces and zero-index materials

Author: Hongchen Chu et al

Publication: Light: Science & Applications

Publisher: Springer Nature

Date: Aug 15, 2018

Copyright © 2018, The Author(s)

Creative Commons

This is an open access article distributed under the terms of the Creative Commons CC BY license, which permits unrestricted use, distribution, and reproduction in any medium, provided the original work is properly cited.

You are not required to obtain permission to reuse this article.

To request permission for a type of use not listed, please contact Springer Nature
