# Supplementary material for: Dirac-like cone-based electromagnetic zero-index metamaterials
Source: Light Sci Appl. 2021 Sep 30;10:203. doi: 10.1038/s41377-021-00642-2 (PMC8481486; doi:10.1038/s41377-021-00642-2)
Supplement: Supplementary file 17 — Permission_Figure6-2016Supercoupler [file 41377_2021_642_MOESM17_ESM.pdf]

Strongly Extended Superradiance in Diamond Metamaterials

Olivia Mello, Yang Li, Phil Camayd-Muñoz, Cleaven Chia, I-Chun Huang , Marko Lončar, Eric Mazur

School of Engineering and Applied Sciences, Harvard University, 9 Oxford Street, Cambridge, Massachusetts 02138

oliviamello@g.harvard.edu

Abstract: Zero index metamaterials (ZIM) experience near-perfect spatial coherence and infinite spatial wavelength. We model, through both analytical calculations and simulations, superradiant emission of silicon vacancy centers (SiV) in a diamond ZIM that extends well beyond the emission wavelength near 737 nm.

OCIS codes: (270.0270) Superradiance, superfluorescence; (160.3918) Metamaterials; (270.6630) Quantum optics

1. Introduction

We have recently demonstrated a metamaterial with zero refractive index (ZIM), which creates a lack of spatial phase advance of light propagating inside it [1]. We can use spatial coherence to obtain perfect Dicke superradiance (Figure 1a) throughout space with low radiative loss in ZIMs [1,2]. From our analysis we anticipate an almost perfectly extended superradiant dipole for distances greater than 10 μm, and therefore propose to use this platform to achieve superradiance and enhanced decay rate (Figure 1b) of many atoms in a highly extended two-dimensional sample.

2. Analytical Results

To solve for the decay rates of N-atom superradiance, we create an ansatz of an N-atom Dicke state and use in the Schrödinger equation. This creates a many-body eigenvalue problem which we can solve to find the superradiant decay rate [3]. The resulting solution for the decay rates for the Dicke states is presented in [3] as a sum of Bessel functions.

Figure 1c presents the cooperative enhancement of decay rate for $N = 1000$ atoms. We experience a massive enhancement in decay rate as the average interatomic distance increases for lower indices of refraction. At $n = 0$, the cooperative enhancement ratio is 1000 throughout the entire operating space of the cloud of atoms regardless of distance (green curve in Figure 1c). With our ability to attain zero refractive index with low linear losses, we can anticipate an almost perfectly superradiant dipole for distances greater than 10 μm.

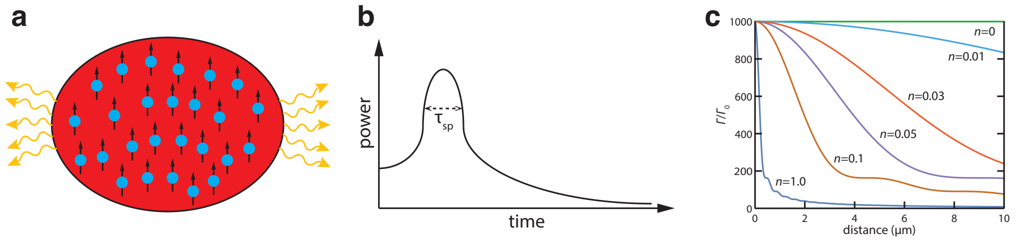

Fig. 1. (a) Coherent superradiant emission with no spatial phase variation. (b) Superradiant decay with characteristic shortened timescale. (c) Superradiant decay rates for varying indices of refraction n in a system of 1000 atoms as a function of average interatomic distance.

3. Simulation and Design

To experimentally demonstrate superradiance of SiVs in a diamond-based nanostructure with a long effective wavelength, we design a diamond-based Dirac-cone metamaterial with an effective index of zero at the emission wavelength of SiV − 737 nm [4] (Figure 2a). Because of a Dirac-cone dispersion at the center of the Brillouin zone, Dirac-cone

metamaterials achieve an impedance-matched effective index of zero as a result of the fact that the effective permittivity and permeability cross zero simultaneously and linearly at the Dirac-point wavelength [5]. As shown in Figure 2b, the effective refractive index of this metamaterial crosses zero at the design wavelength, corresponding to an infinite effective wavelength, allowing the SiVs within the metamaterial emit photons with perfect spatial coherence. This design is an ideal platform for demonstrating superradiance due to following reasons:(1) it can show an isotropic, impedance-matched, low-loss effective index of zero at the emission wavelength of SiV; (2) the SiVs can be integrated into such a structure while maintaining their coherence properties [6]; (3) it can be fabricated through patterning and etching bulk diamond which is much easier than the fabrication of thin single-crystal diamond films [7, 8]; (4) it allows us to pump the metamaterial either or both from the out-of- plane (z axis in Figure 2a) and in-plane (any direction in x-y plane of Figure 2a) directions.

We use full-wave numerical simulations (FDTD) to model the emission from randomly positioned dipoles within a square array of diamond pillars. Dipoles are seeded with an average density $\rho = 45$ dipoles$/\mu m^2$ (10 dipoles per unit cell) to approximate ion-implanted SiVs. The dipole moments are oriented perpendicular to the plane of the array, corresponding to (111)- oriented diamond that is provided by our industry collaborators at Element 6. After coherent excitation, the dipoles radiate in phase throughout the material. Figure 2c shows the total power radiating from the pillar array, for increasing numbers of pillars, n. As more pillars are added, the number of dipoles also increases, where $N \propto \rho n^2$. Due to the long effective wavelength at zero index, these sources emit coherently into the same long-range supermode; as a result, the radiated power increases in proportion to the square of the population N^2, a hallmark of superradiance. In effect, ZIM extends the effective wavelength in the metamaterial such that all of the emitters occupy the same deeply sub-wavelength volume.

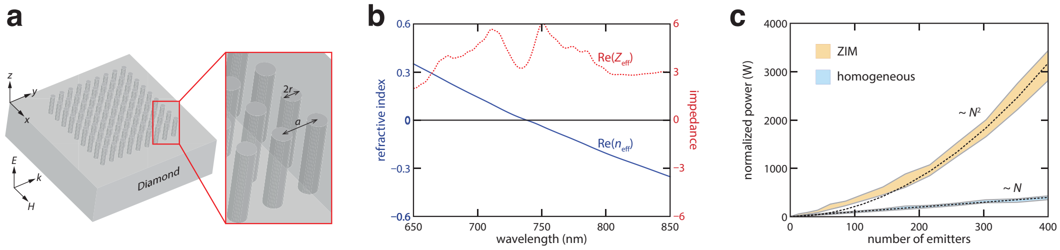

Fig. 2. (a) Dirac-cone-based zero-index metamaterial consisting of a square array of 1μm tall diamond pillars on a diamond substrate for transverse-magnetic (TM) polarization. (b) Effective index and impedance of the optimized Dirac-cone-based zero-index metamaterial. (c) Total radiated power from different-sized arrays. In the metamaterial the power increases quadratically with the number of emitters, N.

References

1. Y. Li, S. Kita, P. Muoz, O. Reshef, D. Vulis, M. Yin, et al., ”On-chip zero-index metamaterials,” Nat. Photon., **9**, pp. 738-742, (2015).
2. R. H. Dicke, ”Coherence in spontaneous radiation processes,” Phys. Rev., **93**, pp. 99-110, (1954).
3. M. O. Scully, E. S. Fry, C. H. R. Ooi, and K. Wódkiewicz, ”Directed spontaneous emission from an extended ensemble of N atoms: timing is everything,” Phys. Rev. Lett., **96**, p. 010501, (2006).
4. S. Kita, Y. Li, P. Muoz, O. Reshef, D. Vulis, B. Day, et al., ”On-chip super-robust all-dielectric zero-index material,”; in CLEO: 2015, San Jose, California, 2015, p. FM3C.2.
5. X. Q. Huang, Y. Lai, Z. H. Hang, H. H. Zheng, and C. T. Chan, ”Dirac cones induced by accidental degeneracy in photonic crystals and zero-refractive- index materials,” Nat. Mater., **10**, pp. 582-586, 2011.
6. A. Sipahigil, R. E. Evans, D. D. Sukachev, M. J. Burek, J. Borregaard, M. K. Bhaskar, et al., ”An integrated diamond nanophotonics platform for quantum-optical networks,” Science, **354**, pp. 847-850, 2016.
7. T. M. Babinec, B. J. M. Hausmann, M. Khan, Y. Zhang, J. R. Maze, P. R. Hemmer, et al., ”A diamond nanowire single-photon source,”; Nat. Nano., **5**, pp. 195-199, 2010.
8. B. J. M. Hausmann, I. Bulu, V. Venkataraman, P. B. Deotare, and M. Loncar, ”Diamond nonlinear photonics,” Nat. Photon., **8**, pp. 369-374, 2014.
