# Supplementary material for: Dirac-like cone-based electromagnetic zero-index metamaterials
Source: Light Sci Appl. 2021 Sep 30;10:203. doi: 10.1038/s41377-021-00642-2 (PMC8481486; doi:10.1038/s41377-021-00642-2)
Supplement: Supplementary file 19 — Permission_Figure6-2017Superradiance [file 41377_2021_642_MOESM19_ESM.pdf]

Phase-Matching in Dirac-Cone-Based Zero-Index Metamaterials

Orad Reshef, Yang Li, Mei Yin, Lysander Christakis, Daryl I. Vulis, Philip Camayd-Muñoz, Shota Kita, Marko Lončar, Eric Mazur

School of Engineering and Applied Sciences, Harvard University, 9 Oxford St, Cambridge, MA 02138, USA

mazur@seas.harvard.edu

Abstract: Using nonlinear scattering theory, we simulate nonlinear signal generation in 2-dimensional zero-index metamaterials based on a photonic Dirac cone at the Γ point. We observe unique phase-matching in multiple simultaneous directions as the index approaches zero.

OCIS codes: (160.3918) Metamaterials; (190.4223) Nonlinear wave mixing.

1. Introduction

Dirac cones at the center of the Brillouin zone have been used to achieve a metamaterial with a refractive index of zero [1, 2, 3]. Previously, simultaneous bi-directional phase-matching behaviour has been demonstrated using an anisotropic zero-index metamaterial (ZIM) [4]. Here, we use nonlinear scattering theory [5, 6] to simulate an isotropic on-chip ZIM based on photonic Dirac cones and demonstrate simultaneous phase-matching in all directions.

2. Photonic Dirac-cone-based ZIM

We investigate two platforms: a 2D square array of silicon pillars in air (pitch = 845 nm, radius = 171 nm) and a 2D square array of airholes in a silicon bulk (pitch = 583 nm, radius = 182 nm). These metamaterials are designed to exhibit a Dirac cone at the center of their respective Brillouin zones (Fig. 1), indicating an effective refractive index of zero.

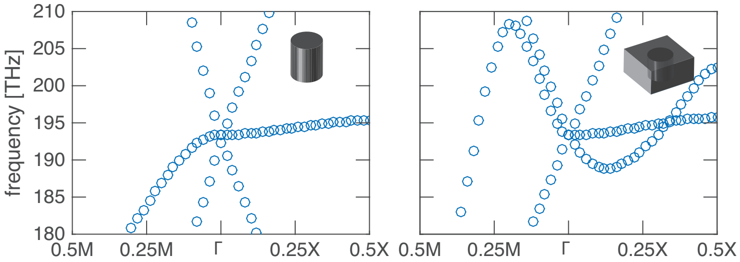

Fig. 1. The band structures for 2D square arrays of rods (left) and airholes (right) are designed to exhibit Dirac cones at the center of the Brillouin zone.

3. Nonlinear scattering theory

Using nonlinear scattering theory, we calculate the relative intensity of the nonlinear signal generated within a medium using $I_{\text{NL}} \propto \left|\chi^{(3)} \int \vec{E} \cdot \vec{P}_{\text{NL}}\, dV\right|^2$ [5]. We verify this method by simulating degenerate four-wave mixing (FWM) within bulk materials with dispersionless refractive indices of $n = 2, 1, 0.5$, and 0.01 (Fig. 2). As expected, the coherence lengths for these interactions are infinite for light that is generated in the forward-propagating direction, regardless of the material index. On the other hand, the coherence length increases in the backward-propagating direction as the index decreases, in good agreement with the predicted value of $L_{\text{coh}} = {}^{\pi c}/_{2\omega n} \approx 400\,\text{nm}/n$.

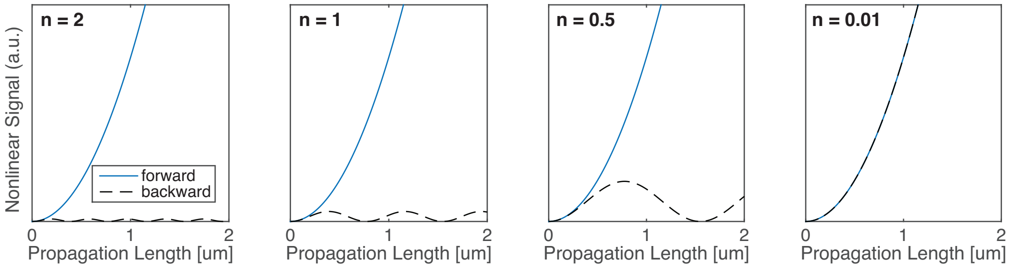

Fig. 2. Nonlinear signals computed using the nonlinear scattering method for dispersionless bulk media, exhibiting different backward-phase-matching behavior as a function of index.

4. Phase-matching in Dirac-cone-based ZIMs

Using the method outlined in Section 3, we calculate the nonlinear signal generated in both the rod-based and airhole-based ZIMs as a function of propagation length (Fig. 3a-b). We normalize the intensities to the peak intensity measured. The generated intensities all grow quadratically in all propagation directions, indicating perfect phase-matching. This result qualitatively resembles the $n = 0.01$ case in Fig. 2. We also observe phase-matching behavior when increasing the size of the ZIM laterally, orthogonally to the direction of the input pump (Fig. 3c).

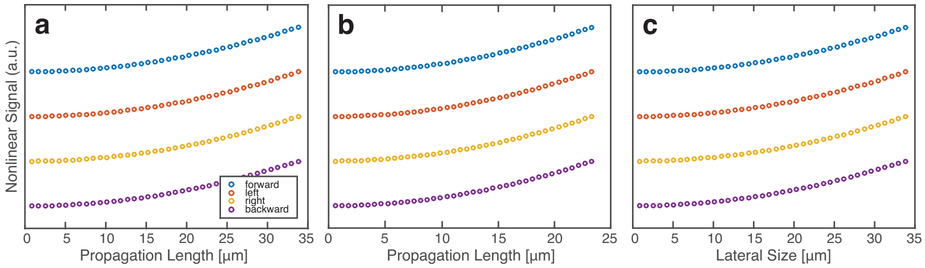

Fig. 3. Nonlinear signals computed for (a) rod-based and (b) airhole-based ZIMs as a function of propagation length. The nonlinear signal also grows quadratically as the device increases in the lateral direction (c).

5. Conclusion

We have used nonlinear scattering theory in Dirac-cone-based ZIMs to demonstrate the simultaneously phase-matched generation of nonlinear signal in multiple directions, consistent with a refractive index of zero. This phase-matched signal generation is independent of shape, ZIM size, and excitation direction.

References

1. Y. Li, S. Kita, P. Muñoz, O. Reshef, D. I. Vulis, M. Yin, M. Lončar, and E. Mazur, "On-chip zero-index metamaterials," Nature Photonics **9**, 738–742 (2015).
2. X. Huang, Y. Lai, Z. H. Hang, H. Zheng, and C. T. Chan, "Dirac cones induced by accidental degeneracy in photonic crystals and zero-refractive-index materials." Nature Materials **10**, 582–586 (2011).
3. P. Moitra, Y. Yang, Z. Anderson, I. I. Kravchenko, D. P. Briggs, and J. Valentine, "Realization of an all-dielectric zero-index optical metamaterial," Nature Photonics **7**, 791–795 (2013).
4. H. Suchowski, K. O'Brien, Z. J. Wong, A. Salandrino, X. Yin, and X. Zhang, "Phase Mismatch-Free Nonlinear Propagation in Optical Zero-Index Materials," Science **342**, 1223–1226 (2013).
5. K. O'Brien, H. Suchowski, J. Rho, A. Salandrino, B. Kante, X. Yin, and X. Zhang, "Predicting nonlinear properties of metamaterials from the linear response," Nature Materials **14**, 379–383 (2015).
6. J. Butet and O. J. F. Martin, "Evaluation of the nonlinear response of plasmonic metasurfaces: Miller's rule, nonlinear effective susceptibility method, and full-wave computation," Journal of the Optical Society of America B **33**, A8–A15 (2016).
