# Supplementary material for: Dirac-like cone-based electromagnetic zero-index metamaterials
Source: Light Sci Appl. 2021 Sep 30;10:203. doi: 10.1038/s41377-021-00642-2 (PMC8481486; doi:10.1038/s41377-021-00642-2)
Supplement: Supplementary file 20 — Permission_Figure6-2018Cloaking_in_free_space [file 41377_2021_642_MOESM20_ESM.pdf]

Design of Dirac-point photonic crystal quantum cascade lasers

Yong Liang[1,*], Zhixin Wang[2], Xuefan Yin[2], Chao Peng[2], Weiwei Hu[2], and Jerome Faist[1]

[1] *Institute for Quantum Electronics, ETH Zürich, Zürich, Switzerland*
[2] *State Key Laboratory of Advanced Optical Communication Systems and Networks, Peking University, Beijing, China*

liangyo@phys.ethz.ch

Abstract: We design photonic-crystals enabling laser operation at the Dirac-point, based on three-dimensional simulations of realistic quantum cascade laser structures. We demonstrate the feasibility of using the Dirac-point mode for higher-power single-mode photonic-crystal quantum cascade lasers.

OCIS codes: 140.5965, 050.5298.

1. Introduction

Photonic-crystal surface-emitting lasers (PCSELs) [1] are semiconductor emitters that are inherently suitable for high-power, large-area and single-mode operation [2], owing to the multidirectional distributed feedback effect provided by photonic crystals (PhCs) [3, 4]. Recently, Dirac-point of a square-lattice air-hole PhC slab [5, 6] was proposed as a promising candidate to further enlarge the lasing area of PCSELs based on interband transitions (TE polarization). At such Dirac-point, the accidental-degeneracy of three band-edge modes leads to a linear dispersion rather than a conventional quadratic one. However, the drawback of such proposal is the difficulty of selecting the Dirac-point for lasing. It was claimed that, among these triply-degenerated Dirac-point modes, the high-$Q_{\perp}$ mode is selected for lasing due to its lowest radiation loss. In fact, for a *finite-size* PCSEL with air-hole PhCs under TE polarization, the other high-$Q_{\perp}$ quadratic-dispersion mode will be selected for lasing because it has the strongest in-plane confinement (highest $Q_{\parallel}$) compared to all the Dirac-point modes. This is critical for Dirac-point laser design, because the advantages of using a Dirac-point will not be obtained. Here, to realize a PCSEL operating at the desirable Dirac-point, we propose to use rod-based PhC structures under TM polarization, which can be implemented by incorporating PhCs into the active-region (AR) of quantum cascades lasers (QCLs) based on intersubband transitions [7]. Via 3D numerical simulations, we show that, our proposed PhC QCL structure ensures lasing at the desirable Dirac-point without being affected by the presence of the quadratic-dispersion mode, therefore providing a feasible approach to realize a Dirac-point laser.

2. Numerical results

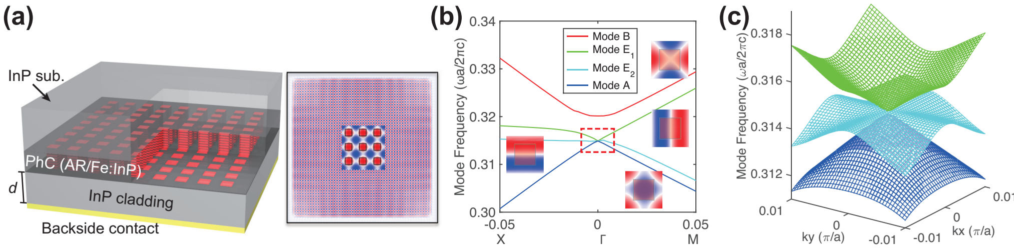

Fig. 1. (a) Schematic of a PhC surface-emission QCLs (left) and single-mode field distribution over a large-area PhC (right). The period of PhC, $a = 2.7\ \mu$m. The AR (red) of QCLs is patterned into a 2D square array of square-shaped PhC pillars by deep dry-etching. Surrounding medium of PhC pillars is Fe-doped InP (dark gray) by regrowth [8]. (b) Band structure (TM-like) designed to exhibit a linear Dirac dispersion. Insets show field patterns of the four modes at the 2nd-order Γ-point. (c) 3D dispersion surfaces nearby the Dirac-point indicated by red dashed rectangle in (b).

Figure 1(a) shows a schematic of a PhC QCL for surface-emission, which features a large-area single-mode lasing in the PC plane. By tuning the size of PhC pillars, a band structure exhibiting a linear Dirac dispersion (green and blue bands) is obtained, as shown in Figs. 1(b) and (c). The monopole mode A and the doubly-degenerate dipole modes $E_{1,2}$ accidentally degenerate at the Γ-point. In contrast, the high-$Q_\perp$ quadrupole mode B (red band) is far away ($\Delta\omega = 5.0\times10^{-3}\,\omega a/2\pi c$) from the accidental-degeneracy and exhibits a quadratic dispersion.

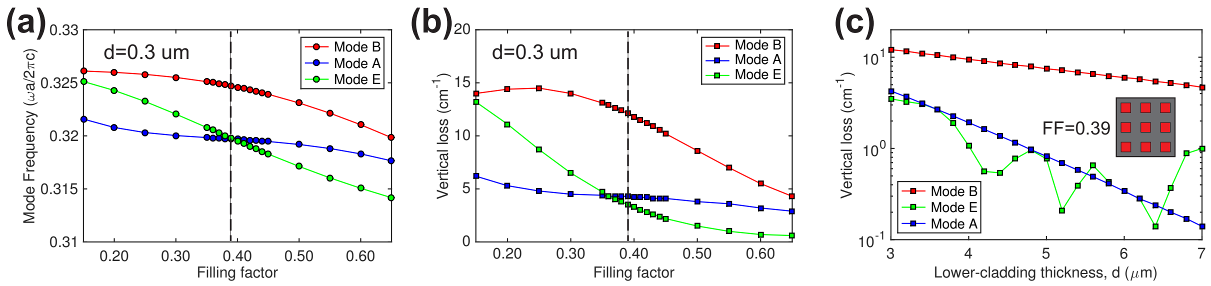

Fig. 2. Frequency (a) and vertical loss (b) of the Γ-point modes versus FF, calculated by COMSOL with a backside metal below the PhCs. The lower InP-cladding thickness, $d = 3.0\ \mu$m [see Fig. 1(a)]. The dashed black line indicates FF$=0.39$, where accidental Dirac-point degeneracy occurs. (c) Vertical loss of the Γ-point modes versus d. The calculations are performed for PhC pillars with FF$=0.39$ (inset), where accidental-degeneracy of the three modes A, E_1 and E_2 is obtained.

Figures 2(a) and (b) show the dependence of mode frequency and vertical loss on PhC pillar size. The vertical loss includes both the radiation loss and waveguide loss induced by the backside metal-contact [see Fig. 1(a)]. At filling factor (FF) =0.39, mode A and doubly-degenerate modes $E_{1,2}$ form the Dirac-point, while their vertical losses are not identical. It is important to notice that, the vertical loss of mode B is $\sim$8 cm^{-1} larger than that of the Dirac-point modes at FF=0.39, therefore preventing mode B from lasing and ensuring laser to operate at the desirable Dirac-point. Next, to tune the vertical loss of the Dirac-point modes (at FF=0.39), we increase the separation distance (d) between PhC layer and the metal, as shown in Fig. 2(c). As expected, the vertical losses of high-$Q_\perp$ monopole mode A and quadrupole mode B both decay exponentially as a function of d. In contrast, vertical loss of the low-$Q_\perp$ dipole modes $E_{1,2}$ [3,4] oscillates with a period of $\sim$0.5a, arising from the reflections at the backside contact [2]. Such oscillation behavior is another advantage of our design, because we can choose a proper d to make both the frequency and modal loss of Dirac-point modes degenerate, simultaneously. On the other hand, we can also select one of the Dirac-point modes (i.e., mode E or A) as a lasing mode.

3. Summary

We have presented a design strategy for realising Dirac-point lasing in realistic PhC QCL structures. The proposed PhC structure suppresses the high-$Q_\perp$ quadratic-dispersion mode lasing and provides a feasible solution for larger-area higher-power single-mode PCSELs operating at the Dirac-point with a flexible mode control.

References

[1] E. Miyai, K. Sakai, T. Okano, W. Kunishi, D. Ohnishi, and S. Noda, "Photonics: lasers producing tailored beams," Nature **441**, 946–946 (2006).

[2] K. Hirose, Y. Liang, Y. Kurosaka, A. Watanabe, T. Sugiyama, and S. Noda, "Watt-class high-power, high-beam-quality photonic-crystal lasers," Nature photonics **8**, 406–411 (2014).

[3] Y. Liang, C. Peng, K. Sakai, S. Iwahashi, and S. Noda, "Three-dimensional coupled-wave model for square-lattice photonic crystal lasers with transverse electric polarization: A general approach," Physical Review B **84**, 195,119 (2011).

[4] Y. Yang, C. Peng, Y. Liang, Z. Li, and S. Noda, "Analytical perspective for bound states in the continuum in photonic crystal slabs," Physical Review Letters **113**, 037,401 (2014).

[5] S.-L. Chua, L. Lu, J. Bravo-Abad, J. D. Joannopoulos, and M. Soljačić, "Larger-area single-mode photonic crystal surface-emitting lasers enabled by an accidental dirac point," Optics Letters **39**, 2072–2075 (2014).

[6] B. Zhen, C. W. Hsu, Y. Igarashi, L. Lu, I. Kaminer, A. Pick, S.-L. Chua, J. D. Joannopoulos, and M. Soljačić, "Spawning rings of exceptional points out of dirac cones," Nature **525**, 354–358 (2015).

[7] J. Faist, F. Capasso, D. L. Sivco, C. Sirtori, A. L. Hutchinson, and A. Y. Cho, "Quantum cascade laser," Science **264**, 553–556 (1994).

[8] R. Peretti, V. Liverini, M. J. Süess, Y. Liang, P.-B. Vigneron, J. M. Wolf, C. Bonzon, A. Bismuto, W. Metaferia, M. Balaji *et al.*, "Room temperature operation of a deep etched buried heterostructure photonic crystal quantum cascade laser," Laser & Photonics Reviews **10**, 843–848 (2016).
