# Supplementary material for: Dirac-like cone-based electromagnetic zero-index metamaterials
Source: Light Sci Appl. 2021 Sep 30;10:203. doi: 10.1038/s41377-021-00642-2 (PMC8481486; doi:10.1038/s41377-021-00642-2)
Supplement: Supplementary file 6 — Permission_Figure3b [file 41377_2021_642_MOESM6_ESM.pdf]

Subject:	RE: Request permission of reusing a figure in a paper published in Optics Letters
From:	pubscopyright <copyright@osa.org> Aug 12, 2021 10:28:07 PM
To:	"李杨" <yli9003@mail.tsinghua.edu.cn>, pubscopyright <copyright@osa.org>

Dear Dr. Li,

Thank you for contacting The Optical Society.

For the use of figure 4 from J. A. Rodríguez, B. Wang, and M. A. Cappelli, "Dual-polarization Dirac cones in a simple 2D square lattice photonic crystal," Opt. Lett. 45, 2486-2489 (2020):

> OSA considers your requested use of its copyrighted material to be Fair Use under United States Copyright Law. It is requested that a complete citation of the original material be included in any publication.

While your publisher should be able to provide additional guidance, OSA prefers the below citation formats:

> For citations in figure captions:
>
> [Reprinted/Adapted] with permission from [ref #] © The Optical Society. (Please include the full citation in your reference list)
>
> For images without captions:
>
> Journal Vol. #, first page (year published) An example: Biomed. Opt. Express 6, 793 (2015)

Please let me know if you have any questions.

Kind Regards,

Hannah Greenwood

Hannah Greenwood

August 12, 2021

Authorized Agent, The Optical Society

The Optical Society (OSA)

2010 Massachusetts Ave., NW

Washington, DC 20036 USA

www.osa.org

Reflecting a Century of Innovation

From: 李杨 <yli9003@mail.tsinghua.edu.cn>
Sent: Thursday, August 12, 2021 4:06 AM
To: pubscopyright <copyright@osa.org>
Subject: Request permission of reusing a figure in a paper published in Optics Letters

Dear OSA Editors:

My name is Yang Li, I am a faculty member of the department precision instrument at Tsinghua University, Beijing. At this moment, I plan to reuse Figure 4 of following paper in our coming review paper with the title "Dirac-like cone-based electromagnetic zero-index metamaterials" which is recently accepted by the journal "Light: Science and Applications".

J. A. Rodríguez, B. Wang, and M. A. Cappelli, "Dual-polarization Dirac cones in a simple 2D square lattice photonic crystal," Optics Letters, vol. 45, no. 9, pp. 2486-2489, 2020.

Could you please give me instruction on how to reuse this figure in our review paper? Thank you!

Best regards,

Yang

--

李杨

副教授

清华大学，精密仪器系

通讯地址：北京市，海淀区，清华大学，9003大楼301-1

手机号：16601021689

电子邮箱：yli9003@mail.tsinghua.edu.cn; 20002000.leon@gmail.com

网页：http://faculty.dpi.tsinghua.edu.cn/yli9003.html (系个人主页)

http://yligroup.com/ （课题组）

Yang Li

Associate Professor

The Department of Precision Instrument, Tsinghua University

Yang Li

Room 301-1, 9003 Building

Tsinghua University

Haidian District

Beijing, China 100084

Tel: +86.16601021689 (Mobile)

E-mail: yli9003@mail.tsinghua.edu.cn; 20002000.leon@gmail.com

Website: http://faculty.dpi.tsinghua.edu.cn/en_yli9003.html (department profile)

http://yligroup.com/ (group)
