# Supplementary material for: Dirac-like cone-based electromagnetic zero-index metamaterials
Source: Light Sci Appl. 2021 Sep 30;10:203. doi: 10.1038/s41377-021-00642-2 (PMC8481486; doi:10.1038/s41377-021-00642-2)
Supplement: Supplementary file 14 — Permission_Figure5jk [file 41377_2021_642_MOESM14_ESM.pdf]

Subject:	Re: Request permission of using a figure of your CLEO proceeding	
From:	"梁永" <lian21.great@gmail.com>	Aug 12, 2021 2:43:27 PM
To:	"李杨" <yli9003@mail.tsinghua.edu.cn>	

Dear Prof. Li,

Thanks for showing interest in our work.

I hereby give you permission to reuse the Figure1a of our CLEO proceeding (2017) that you mentioned below.

Best regards,
Yong Liang
Sent from my iPhone

> On 12. Aug 2021, at 05:10, 李杨 <yli9003@mail.tsinghua.edu.cn> wrote:
>
> Dear Dr. Liang:
>
> Currently, I am finalizing a review article on the topic of "Dirac-like cone-based electromagnetic zero-index metamaterials" and would like to reuse Figure 1a of your following CLEO proceeding.
>
> Y. Liang, Z. Wang, X. Yin, C. Peng, W. Hu, and J. Faist, "Design of Dirac-point photonic crystal quantum cascade lasers," in Conference on Lasers and Electro-Optics, San Jose, California, 2017, p. JTu5A.112: Optical Society of America.
>
> At the position of this reusing, I will cite your CLEO proceeding properly. Could you please give me the permission to reuse this figure? Thank you!
>
> Best regards,
>
> Yang
>
> --

> 李杨
> 副教授
> 清华大学，精密仪器系
>
> 通讯地址：北京市，海淀区，清华大学，9003大楼301-1
> 手机号：16601021689
> 电子邮箱：yli9003@mail.tsinghua.edu.cn; 20002000.leon@gmail.com
> 网页：http://faculty.dpi.tsinghua.edu.cn/yli9003.html (系个人主页)
> http://yligroup.com/ （课题组）
>
> Yang Li
> Associate Professor
> The Department of Precision Instrument, Tsinghua University
>
> Yang Li
> Room 301-1, 9003 Building
> Tsinghua University
> Haidian District
> Beijing, China 100084
> Tel: +86.16601021689 (Mobile)
> E-mail: yli9003@mail.tsinghua.edu.cn; 20002000.leon@gmail.com
> Website: http://faculty.dpi.tsinghua.edu.cn/en_yli9003.html (department profile)
> http://yligroup.com/ (group)
